# The association between blood heavy metals level and sex hormones among postmenopausal women in the US

**DOI:** 10.3389/fendo.2023.1175011

**Published:** 2023-07-18

**Authors:** Wenchao Zhang, Yugui Cui, Jiayin Liu

**Affiliations:** State Key Laboratory of Reproductive Medicine, Clinical Center of Reproductive Medicine, The First Affiliated Hospital, Nanjing Medical University, Nanjing, China

**Keywords:** environmental exposure to heavy metals, gonadal hormones, postmenopause, National Health and Nutrition Examination Survey, cross-sectional study

## Abstract

**Introduction:**

Environmental pollutants could be implicated in female endocrine setting Q6 beyond traditional factors. Until now, few study has focused on the association of environmental exposure to heavy metals with sex hormones in postmenopausal women. This study intended to investigate whether serum levels of heavy metals(i.e., Cd, Pb, Hg, Mn, Se) would influence sex hormones in postmenopausal women.

**Methods and results:**

A cross-sectional study was performed on 614 nationally representative participants from 2013-2016 National Health and Nutrition Examination Survey (NHANES) in the US. Multivariate linear regression models and restricted cubic spline plots revealed cadmium(Cd) had linear positive association with TT(β=3.25, 95%CI= 1.12, 5.38), bioavailable TT(β=1.78, 95%CI=0.36,3.21) and TT/E2(β=0.76, 95%CI=0.28,1.24), which was more apparent in natural menopausal and obese women. Lead(Pb) had linear positive association with SHBG(β=12.84, 95%CI= 6.77,18.91), which was apparent in nearly all subgroups except in normal BMI group, and TT/E2 (β=0.69, 95%CI 0.134,1.25), which was apparent in natural menopausal and normal BMI women. Manganese(Mn) had non-linear association with SHBG, which was more apparent in natural menopausal and obese women, and TT/E2, which was more apparent in natural menopausal and normal BMI women. Selenium(Se) had U shaped non-linear association with TT, which was more apparent in hysterectomy, overweight and obese women, and SHBG, which was apparent in nearly all subgroups except in normal BMI group.

**Conclusion:**

In summary, this cross-sectional study indicates a possible role that various degree of environmental exposure to heavy metals plays in the disruption of sex Q5 hormone levels in postmenopausal women. Further experiments are needed to elucidate the underlying mechanisms.

## 1 Introduction

Sex hormones play a major role in regulating female reproduction. Total testosterone(TT), estradiol (E2), and sex hormone-binding globulin (SHBG) are three main sex hormones in the human body (1). TT is synthesized in much smaller amounts, in the ovary and the adrenal gland of female. It is widely acknowledged that elevated serum TT not only causes virilizing effects in postmenopausal women, but also will lead to hypercholesterolemia, insulin resistance, hypertension and cardiac disease (2, 3). E2 is produced primarily in the ovary, but small quantities are also formed in the adrenal cortex, as well as fat cells. Impairment of the E2 secretion has been found to be associated with many reproductive disorders, including anovulation, premature ovarian failure and even amenorrhea (4, 5). SHBG is produced and secreted by liver into the blood where it binds sex steroids and regulates their bioavailability (6). Elevated SHBG levels are often found in patients with hyperthyroidism, oral contraceptives or antiepileptic drugs taken and pregnant women (7). Moreover, SHBG could also serve as a biomarker of the degree of inflammation in metabolic diseases (8). Stacks of environmental factors may disturb the levels of TT, E2 and SHBG, which could prompt adverse outcomes in the human body. By the time women are in postmenopausal period, TT and E2 level would drop gradually physiologically with age.

On a daily basis, human beings are unintentionally exposed to heavy metals to varying degrees, which are a class of pollutants that globally exist in air, water, food and soil (9). Numerous studies (9, 10) support that massive doses of exposure to heavy metals have been found to induce oxidative stress, DNA damage and carcinogenicity on a variety of body tissues and organs and bioaccumulation of these heavy metals would disrupt the function of endocrine and reproductive systems (11–13). Conventional heavy metals consist of lead(Pb), mercury(Hg), Cadmium(Cd), Manganese(Mn) and selenium(Se). Elevated levels of Pb and Cd are most commonly caused by occupational exposure. Conversely, Hg bioaccumulates mainly through food consumption, particularly the consumption of fish containing methyl mercury. Mn is a key component of low-cost stainless steel and certain aluminum alloys, which are frequently encountered in daily life. Trace amounts of Se are necessary for cellular function in all animals and are primarily obtained through food consumption. However, excessive exposure to Se and Mn, which is often released by industries, can lead to toxicity in the human body.

Accumulating evidence has demonstrated that the varying degree of heavy metals exposure might exert diverse effect on sex hormone levels in diverse population and at different life stages, which renders it valuable to establish a clear and explicit relationship (14). Previous studies have demonstrated that exposure to arsenic (As), lead (Pb), chromium (Cr), nickel (Ni), urinary phthalate metabolites and phthalates during pregnancy in Iranian women may have a negative impact on the health of their children (15–18). Telisman et al. found a potential synergistic effect between Cd and Pb on increasing serum testosterone levels in men (19). Urinary Hg concentration was found to be associated with lower E2 levels, and urinary Pb concentration was associated with lower E2 and higher follicle-stimulating hormone (FSH) levels in midlife women (20). Nagata’s study (21) indicated inverse associations between urinary Cd and the plasma E2 or TT level in pre- and post-menopausal Japanese women. Considering the current evidence of postmenopausal women is limited, it is of great demand to conduct further research in order to fully understand and describe the changing trends between blood heavy metal levels, including Pb, Hg, Cd, Mn and Se, and sex hormones in postmenopausal women, which will provide some insight into the current study for clinical implications.

The purpose of the present cross-sectional study is to explore the impact of the exposure of heavy metals on sex hormones in postmenopausal women based on the nationally representative population extracted from 2013-2014 and 2015-2016 NHANES. For the first time, the study calculated bioavailable TT and E2 using equations based on the mass action law, which were adopted as outcome variables for analysis, given the fact that most of the circulating TT and E2 are bound to carrier proteins (i.e., SHBG and albumin). Besides, subgroup analyses was performed stratified by type of menopause and body mass index(BMI), as sex hormone concentration may differ in these groups and obesity may affect steroid hormone conversion. Moreover, sensitivity analysis was conducted to ensure the stability of our results with sample weights. By employing univariate and multivariate linear regression models, as well as linear and restricted cubic spline models, this study will shed new light on the associations and trends between heavy metal exposure and sex hormones in postmenopausal women.

## 2 Materials and methods

### 2.1 Data source and sample

The analytic sample was retrieved through The National Health and Nutrition Examination Survey (NHANES), conducted by the National Center for Health Statistics (NCHS) to assess the health and nutritional status of adults and children in the United States. A combination of interviews, physical examinations and laboratory tests is included in the survey. The survey examines a nationally representative sample of about 5,000 persons each year in counties across the country. A mobile exam center (MEC) visits 15 counties each year. NHANES also oversamples persons 60 years of age and older, African Americans, and Hispanics, which renders statistic reliability. To achieve statistic validity, the computer-assisted personal interviews (CAPI) system is programmed with built-in consistency checks to reduce data entry errors and it also uses online help screens to assist interviewers in defining key terms used in the questionnaire. Study protocols for NHANES were approved by the NCHS ethnics review board (22). All the participants signed the informed consent before participating in the study. This study extracted data from 2 NHANES year cycles(i.e., 2013-2014, 2015-2016) (n = 20146). After excluding those with missing sex hormone measurements (N=8793), had regular periods in past 12 months (N=2913) or missing corresponding information (N=7892), breast feeding or pregnancy(N=28) and other reason (N=139), had both ovaries removed(N=529), ever use hormone drugs(N=395) and missing data for blood concentration of heavy metals(N=680), the final analysis enrolled 614 participants with complete data on sex hormones(i.e., serum E2, TT, SHBG), blood concentration of heavy metals(i.e., Pb, Hg, Cd, Mn, Se) and other covariates.

### 2.2 The definitions of exposure and outcome variables

The blood concentration of heavy metals(i.e., Pb, Hg, Cd, Mn, Se) was considered as exposure variable. The Cd, Mn, Hg, Pb, and Se content of whole blood specimens was measured using mass spectrometry after a simple dilution sample preparation step. Firstly, during the sample dilution step, a small volume of whole blood is extracted from a larger specimen and mixed to create a uniform distribution of cellular components. This mixing is crucial as certain metals, such as Pb, are primarily associated with red blood cells. Samples with clots or micro-clots are identified and excluded from analysis due to concerns of sample inhomogeneity. The diluted blood samples are prepared by combining 1 part sample, 1 part water, and 48 parts diluent. The diluent contains chemicals that release metals from red blood cells, reduce ionization suppression, prevent clogging, and enable the use of internal standards. Subsequently, the diluted samples are introduced into the mass spectrometer *via* an inductively coupled plasma (ICP) ionization source. The liquid blood sample is converted into aerosol droplets, which are vaporized, atomized, and ionized in a plasma region. The resulting ions, along with argon, enter the mass spectrometer for analysis. The dynamic reaction cell (DRC) plays a crucial role in selective reactions, either eliminating interferences or enhancing the ion signal for specific elements. The ions passing through the DRC are electrically selected and directed to the analytical quadrupole. The electrical signals generated by the ions striking the detector are processed into digital information, allowing for the determination of elemental concentrations. Further details can be found in the Laboratory Method Files section (23). For analytes with analytic results below the lower limit of detection(LLOD), an imputed fill value was placed in the analyte results field, which is the lower limit of detection divided by the square root of 2 (LLOD/sqrt (2)). The LLOD of Pb, Hg, Cd, Se and Mn were 0.07 µg/L, 0.28μg/L, 0.1 μg/L, 24.48 μg/L and 0.99 μg/L, respectively(‘ug/L’ stands for micrograms per liter, It indicates the amount of a substance in micrograms (a unit of mass) per liter (a unit of volume). Subsample weights were adopted in the sensitivity analysis under a complex multistage probability sampling design.

Sex hormones were regarded as outcome variables, including TT(ng/dL: The unit ng/dL stands for nanograms per deciliter. It is a unit of measurement used to report the concentration of testosterone in blood.), E2(pg/mL: The unit pg/mL refers to picograms per milliliter, which indicates the number of picograms (trillionths of a gram) of estradiol present in one milliliter of blood.), SHBG(nmol/L stands for nanomoles per liter, which indicates the number of nanomoles of SHBG per liter of blood.), free testosterone (FT, ng/dL), free estradiol(FE2, pg/mL), TT/E2. Measurements of TT and E2 in serum were performed using isotope dilution liquid chromatography tandem mass spectrometry (ID-LC-MS/MS) method (24). SHBG measurement was based on the reaction of SHBG with immuno-antibodies and chemo-luminescence measurements of the reaction products that occurs after two incubation periods and subjecting to a magnetic field. The lowest detection limits of the assays and the coefficients of variation for quality control specimens were reported previously (25, 26). TT/E2 were defined as the ratio of TT to E2. FT and FE2 were defined as the “bioavailable fraction”, which was defined using equations based on the mass action law (27). FT was calculated from TT, albumin, and SHBG concentrations, FE2 was calculated from E2, albumin, and SHBG concentrations.

### 2.3 The definition of menopausal status

**Figure 1:**
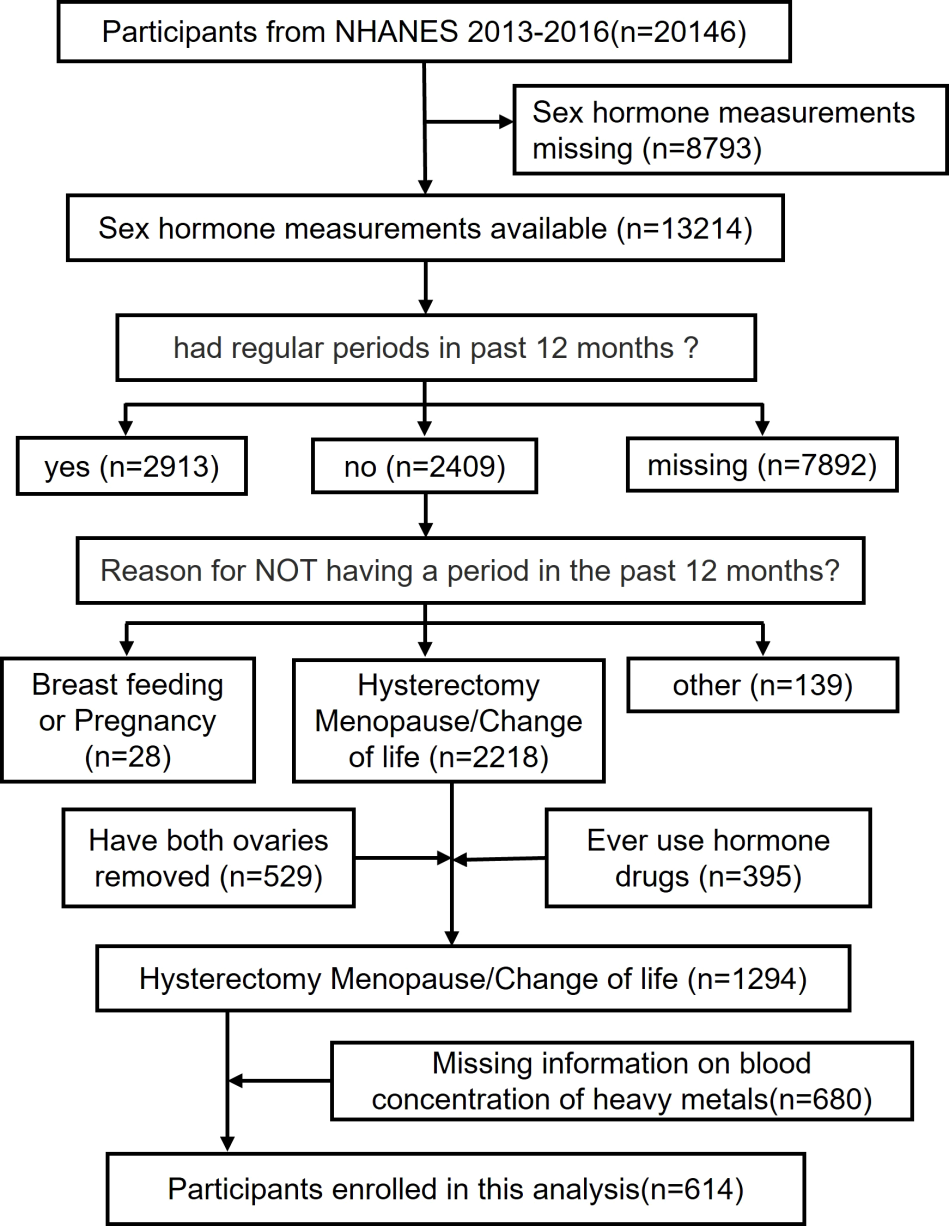
Flow chart of the selection criteria of the study population.

Self-reported information from the Reproductive Health Questionnaire (variable name: RHQ031) (28) was used to define menopausal status. The participant was denoted as postmenopausal if she responded “no” to the question “Have you had at least one menstrual period in the past 12 months?” and subsequently answered “hysterectomy” or “menopause/change of life” to the question “What is the reason that you have not had a period in the past 12 months?”. Another 395 postmenopausal women were excluded due to a history of sex hormone medication use and 529 because of oophorectomy. An additional 680 participants with missing data on blood concentration of heavy metals was also excluded. In the end, 614 postmenopausal women were enrolled in our analysis (**Figure 1**).

### 2.4 Covariates

In order to offer more precise estimate for the association between blood concentration of heavy metals and sex hormones in postmenopausal women, some parameters related to sex hormones (29–31) were adopted and controlled as covariates. Demographic parameters consisted of age, ethnicity (non-Hispanic White, non-Hispanic Black, Mexican American and other race), education level (below high school, high school and above high school), poverty-income ratio, marital status(married or with partners, widowed or divorced, and unmarried). Other covariates included BMI, alcohol user (criteria: ≥2 drinks per day for females, ≥3 drinks per day for males or binge drinking ≥2 days per month), smoker (criteria: smoked over 100 cigarettes in life), physical activity (never, moderate, and vigorous), energy intake, parity, time since menopause, type of menopause(natural menopause and hysterectomy) and time of blood collection(morning, afternoon, evening).

### 2.5 Statistical analysis

In our study, categorical variables were described by percentages, while continuous variables were described by means (standard deviations). To compare the differences in baseline characteristics between natural menopause and hysterectomy women, the chi-square test (for categorical variables) and Student’s t-test (for continuous variables) were employed. Given their skewed distribution, blood concentration of heavy metals was log2-transformed and then divided into four groups, with the lowest quantile as reference.

Unadjusted model and adjusted linear regression models(adjusting for all the characteristics presented in **Table 1**) were applied to evaluate the effect of the blood concentration of heavy metals on TT, E2, SHBG, FT, FE2, and TT/E2. Akaike’s Information Criterion (AIC) was used to determine which model fits the data better (32). Each blood concentration of heavy metals (quantile) was used as an ordinal variable for tests for trend. Restricted cubic spline plots with 4 knots located at the 5th, 35th, 65th and 95th percentiles (the number of knots was selected according to AIC) were generated to present the visual relationships between the blood concentration of heavy metals and sex hormones, and Wald χ2 test was used to test nonlinearity. If AIC value of the restricted cubic spline model was smaller than the linear model, the former was regarded as the better-fitted model to describe the association of exposure with outcome variables. Moreover, if nonlinearity was detected, we applied a two-piecewise logistic regression model to examine the threshold effect of the log2 transformation of the blood concentration of heavy metals on sex hormones to calculate the inflection point.

**Table 1:** Enrolled participant’s characteristics in NHANES 2013–2016 (N =614).

Characteristics	Overall population	Natural Menopause (n=480)	Hysterectomy (n=134)	p
Age,years (mean (SD))	62.52 (10.29)	63.55 (9.23)	58.83 (12.78)	<0.001
Ethnicity, n (%)				0.006
Non-Hispanic White	208 (33.9)	168 (35.0)	40 (29.9)	
Non-Hispanic Black	112 (18.2)	74 (15.4)	38 (28.4)	
Mexican American	123 (20.0)	97 (20.2)	26 (19.4)	
Other race	171 (27.9)	141 (29.4)	30 (22.4)	
Education level, n (%)				0.018
below high school	194 (31.6)	156 (32.5)	38 (28.4)	
high school	153 (24.9)	129 (26.9)	24 (17.9)	
above high school	267 (43.5)	195 (40.6)	72 (53.7)	
Marital status, n (%)				0.342
married or with partners	309 (50.3)	244 (50.8)	65 (48.5)	
widowed or divorced	248 (40.4)	188 (39.2)	60 (44.8)	
unmarried	57 (9.3)	48 (10.0)	9 (6.7)	
Poverty (mean (SD))	2.21 (1.52)	2.21 (1.53)	2.24 (1.47)	0.833
BMI, n (%)				0.342
Normal (<25)	144 (23.5)	244 (50.8)	65 (48.5)	
Overweight (25~29.9)	169 (27.6)	188 (39.2)	60 (44.8)	
Obese (>30)	299 (48.9)	48 (10.0)	9 (6.7)	
Time since last menstrual period (mean (SD))	15.33 (11.00)	14.26 (10.03)	19.12 (13.25)	<0.001
Parity (mean (SD))	2.99 (2.10)	2.90 (2.12)	3.30 (1.99)	0.052
Smoker = yes, n (%)	90 (14.7)	67 (14.0)	23 (17.2)	0.43
Alcohol user= yes, n (%)	303 (49.3)	219 (45.6)	84 (62.7)	0.001
Recreational activity, n (%)				0.04
no	378 (61.6)	307 (64.0)	71 (53.0)	
moderate	223 (36.3)	165 (34.4)	58 (43.3)	
vigorous	13 (2.1)	8 (1.7)	5 (3.7)	
Energy intake (kcal) (mean (SD))	1614.06 (592.70)	1596.91 (595.80)	1676.07 (579.81)	0.218
Time of blood sample collection, n (%)				0.227
morning	310 (50.5)	251 (52.3)	59 (44.0)	
afternoon	223 (36.3)	169 (35.2)	54 (40.3)	
evening	81 (13.2)	60 (12.5)	21 (15.7)	
Sex hormones, Median (Interquartile range)				
Estrogen, E2 (pg/ml))	6.15 (3.50,11.27)	5.88 (3.37,9.88)	8.84 (3.99,30.40)	<0.001
Total testosterone, TT (ng/dl)	17.80 (12.60,27.20)	18.20 (12.97, 27.65)	16.60 (11.62,25.90)	0.12
Sex hormone-binding globulin, SHBG (nmol/l)	56.31 (38.46,85.71)	56.11 (38.45, 84.96)	58.49 (38.64,88.04)	0.95
Blood concentrations of heavy metals, Median (Interquartile range)				
Lead (ug/L)	1.20 (0.79,1.74)	1.24 (0.83,1.78)	1.12 (0.74, 1.62)	0.017
Mercury (ug/L)	0.74 (0.44,1.53)	0.76 (0.45, 1.63)	0.70 (0.43, 1.18)	0.055
Cadmium (ug/L)	0.38 (0.24,0.66)	0.41 (0.25, 0.70)	0.30 (0.20, 0.51)	0.53
Manganese (ug/L)	9.60 (7.69,11.62)	9.73 (7.84,12.05)	9.15 (7.22, 10.48)	<0.001
Selenium (ug/L)	192.80 (178.59, 208.64)	192.80 (178.31, 209.71)	192.88 (179.66, 204.49)	0.51

Subgroup analysis was conducted to evaluate the effect modification by type of menopause (natural menopause, hysterectomy) and BMI (BMI<25kg/m2, BMI=25-29.9kg/m2, BMI≥30kg/m2). Steroid hormone concentration would be different in natural menopause and hysterectomy, and moreover, obesity would affect conversion of steroid hormones (33, 34). For these reasons, the above factors were selected as grouping variables. Sensitivity analysis was performed to reanalyze the dataset and test the stability of our main results with sample weights. The statistical significance was determined by the P-value less than 0.05(two-tailed). All analyses were conducted by R^®^ statistics(v.4.1.0).

## 3 Results

### 3.1 Baseline characteristics of the study participants

A total of 614 postmenopausal women were included in this study from 2013-2016 NHANES. Baseline characteristics of the study population were listed in **Table 1**. As **Table 1** showed, the women who underwent natural menopause tended to have older ages, higher proportion of alcohol user and lower estrogen concentration, while no significant differences were found in education, ethnicity, marital status, poverty, BMI, smoking, time of blood sample collection and energy intake(all P > 0.05) between those women who had natural menopause and who underwent hysterectomy.

### 3.2 Association between the blood concentrations of heavy metals and sex hormones

Linear regression analyses for the association between blood concentrations of heavy metals and sex hormones among postmenopausal women were demonstrated in **Table 2**. As for the crude models, the concentrations of E2 and fE2 showed a statistically significant negative association between blood concentration of Pb(β= -17.64, 95% CI = (-23.18, -12.09) for E2; β= -15.05, 95% CI = (-19.53,-10.56) for fE2), Cd(β=-6.23, 95% CI =(-10.58,-1.89) for E2; β=-5.97, 95% CI =(-9.49,-2.46) for fE2), Mn(β=-15.06, 95% CI = (-25.14,-4.98) for E2; β=-11.90, 95% CI =(-20.0,-3.73) for fE2) and Se(β=-27.72, 95% CI =(-54.00,-1.45) for E2, insignificant for fE2), and had a significant decreasing trend (all P < 0.05 for trend) across the quantile of the concentration of those aforementioned heavy metals. TT, SHBG concentration and TT/E2 were only found to have significant positive association with Pb(β=2.49, 95% CI = (0.079,4.91) for TT; β=16.75, 95% CI = (11.76,21.74) for SHBG; β=1.72, 95% CI = (1.31, 2.13) for TT/E2) and Cd(β=3.07, 95% CI = (1.22, 4.91) for TT; β=10.98, 95% CI =(7.12,14.83) for SHBG; β=1.23, 95% CI = (0.92,1.55) for TT/E2), and had a significant increasing trend (all P < 0.05 for trend) across the quantile of Pb and Cd concentration.

**Table 2:** Relationship Between blood concentrations of heavy metals and sex hormones in postmenopausal women (unweighted).

metal		E2 β(95%CI)		fE2 β(95%CI)		TT β(95%CI)		fTT β(95%CI)		SHBG β(95%CI)		TT/E2 β(95%CI)	
		Crude Model	Adjusted Model	Crude Model	Adjusted Model	Crude Model	Adjusted Model	Crude Model	Adjusted Model	Crude Model	Adjusted Model	Crude Model	Adjusted Model
Lead	Continues	-17.64 (-23.18, -12.09)***	-5.60(-13.34, 2.12)	-15.05(-19.53,-10.56)***	-5.06(-11.33,1.21)	2.49(0.079,4.91)*	1.43 (-1.07, 3.94)	0.71(-1.08, 2.50)	0.03(-1.64, 1.70)	16.75 (11.76,21.74)***	12.84 (6.77,18.91)***	1.72(1.31, 2.13)***	0.69(0.13,1.25)*
	Quantile 1	Reference	Reference	Reference	Reference	Reference	Reference	Reference	Reference	Reference	Reference	Reference	Reference
	Quantile 2	-21.59(-30.81,-12.37)***	-11.56(-23.07,-0.06)*	-18.34(-25.79,-10.90)***	-10.33(-19.66,-1.00)*	-0.24(-4.26,3.76)	0.69(-3.03,4.42)	-0.52(-3.50,2.45)	0.10(-2.38,2.59)	6.57(-1.77,14.92)	4.59(-4.48,13.68)	1.16(0.47,1.85)***	0.59(-0.24,1.42)
	Quantile 3	-24.80(-34.01 -15.60)***	-9.44(-21.27,2.38)	-21.11(-28.55,-13.68)***	-8.54(-18.14,1.04)	-0.29(-4.30,3.71)	-0.93(-4.77,2.90)	-0.92(-3.90,2.05)	-1.13(-3.70,1.42)	11.29(2.95,19.63)**	6.47(-2.86,15.81)	1.52(0.83,2.21)***	0.13(-0.72,0.98)
	Quantile 4	-27.49(-36.73,-18.26)***	-11.97(-24.37,0.42)	-23.39(-30.85,-15.94)***	-10.53(-20.59,-0.48)*	4.48 (0.46,8.50)*	2.76(-1.25,6.79)	1.65(-1.33,4.63)	0.48(-2.19,3.17)	24.78(16.42,33.15)***	17.95(8.16,27.73)***	2.67(1.98,3.36)***	1.28(0.38,2.19)**
	P for trend	<0.001***	0.11	<0.001***	0.083	0.041 *	0.29	0.35	0.95	<0.001***	<0.001***	<0.001***	0.021 *
Mercury	Continues	-1.39(-4.97,2.17)	-0.53 (-5.26, 4.19)	-1.14(-4.04,1.74)	-0.56(-4.40,3.27)	-1.07(-2.59, 0.43)	-0.50(-2.04, 1.02)	-0.75(-1.88,0.36)	-0.34(-1.36,0.67)	2.46(-0.75,5.68)	0.12(-3.64,3.90)	0.13(-0.13,0.40)	0.006(-0.33, 0.35)
	Quantile 1	Reference	Reference	Reference	Reference	Reference	Reference	Reference	Reference	Reference	Reference	Reference	Reference
	Quantile 2	-1.29(-10.82,8.23)	0.14(-11.05,11.35)	-1.29(-10.82,8.23)	0.21(-8.88,9.30)	-1.29(-10.82,8.23)	-2.07(-5.71,1.56)	0.17(-2.82,3.17)	-1.11(-3.53,1.30)	-7.63(-16.20,0.94)	-7.89(-16.79,1.01)	-0.11(-0.83,0.60)	-0.18(-0.99,0.63)
	Quantile 3	5.10(-4.48,14.68)	6.81(-4.60,18.22)	5.10(-4.48,14.68)	4.53(-4.73,13.79)	5.10(-4.48,14.68)	-1.52(-5.23,2.17)	-2.03(-5.04,0.98)	-1.21(-3.68,1.25)	2.35(-6.26,10.97)	2.14(-6.93,11.21)	-0.48(-1.20,0.24)	-0.63(-1.46,0.20)
	Quantile 4	-4.28(-13.87,5.30)	-1.84(-13.97,10.28)	-4.28(-13.87,5.30)	-1.74(-11.59,8.10)	-4.28(-13.87,5.30)	-1.44(-5.38,2.49)	-1.44(-4.46,1.57)	-0.91(-3.53,1.71)	3.21(-5.40, 11.83)	-2.50(-12.14,7.13)	0.35(-0.36,1.08)	0.08(-0.80,0.96)
	P for trend	0.67	0.89	0.61	0.99	0.15	0.55	0.17	0.49	0.14	0.80	0.53	0.82
cadmium	Continues	-6.23(-10.58, -1.89)**	-3.75(-10.37, 2.86)	-5.97(-9.49,-2.46)***	-3.87(-9.23,1.49)	3.07(1.22,4.91)**	3.25(1.12,5.38)**	1.39(0.026,2.76)*	1.78(0.36,3.21)*	10.98(7.12,14.83)***	6.08(0.82,11.33)*	1.23(0.92,1.55)***	0.76(0.28,1.24)**
	Quantile 1	Reference	Reference	Reference	Reference	Reference	Reference	Reference	Reference	Reference	Reference	Reference	Reference
	Quantile 2	-15.31(-24.61,-6.00)**	-9.58(-20.96,1.79)	-13.53(-21.04,-6.01)***	-9.18(-18.41,0.03)	3.76(-0.21,7.74)	1.81(-1.87,5.49)	2.30(-0.64,5.26)	0.91(-1.53,3.37)	9.33(1.04,17.63)*	4.52(-4.55,13.59)	0.97(0.28,1.66)**	4.52(-4.55,13.59)
	Quantile 3	-18.03(-27.76,-8.30)***	-11.40(-23.62,0.81)	-16.41(-24.27,-8.54)	-10.53(-20.43,-0.63)*	3.79(-0.36,7.95)	3.28(-0.67,7.24)	1.34(-1.74,4.43)	2.02(-0.61,4.66)	19.86(11.19,28.54)***	4.34(-5.39,14.09)	1.78(1.06,2.50)***	4.34(-5.39,14.09)
	Quantile 4	-14.83(-24.31,-5.36)**	-8.26(-21.96,5.43)	-13.79(-21.45,-6.14)***	-8.03(-19.14,3.06)	5.60(1.55,9.65)**	5.03(0.59,9.47)*	2.50(-0.50,5.51)	2.50(-0.45,5.46)	21.90(13.46,30.35)***	10.78(-0.14, 21.71)	2.41(1.71,3.11)***	10.78(-0.14,21.71)
	P for trend	0.0031 **	0.19	<0.001***	0.13	0.011 *	0.021*	0.18	0.068	<0.001***	0.076	<0.001***	0.021*
manganese	Continues	-15.06(-25.14, -4.98)**	-8.22(-20.81,4.36)	-11.90(-20.0,-3.73)**	-6.46(-16.68,3.74)	0.23(-4.07,4.54)	0.75(-3.32, 4.84)	0.15(-3.03,3.34)	-0.12(-2.85,2.59)	-1.25(-10.40,7.89)	5.99(-4.042,16.03)	-0.033(-0.79,0.73)	-0.46(-1.38,0.45)
	Quantile 1	Reference	Reference	Reference	Reference	Reference	Reference	Reference	Reference	Reference	Reference	Reference	Reference
	Quantile 2	-2.26(-11.70,7.18)	4.38(-6.38,15.16)	-1.45(-9.11,6.20)	3.68(-5.06,12.42)	3.01(-1.01,7.04)	1.15(-2.35,4.65)	2.92(-0.057,5.90)	0.91(-1.42,3.24)	-4.52(-13.07,4.03)	0.39(-8.23,9.01)	-0.71(-1.43,-0.0037)*	-0.96(-1.75,-0.18)*
	Quantile 3	-11.19(-20.72,-1.66)*	-6.38(-17.87,5.10)	-8.51(-16.24,-0.78)*	-4.81(-14.14,4.50)	0.27(-3.79,4.33)	0.74(-2.99,4.48)	0.62(-2.38,3.62)	0.50(-1.98,2.99)	-5.98(-14.62,2.64)	-1.03(-10.23,8.16)	-0.20(-0.93,0.51)	-0.36(-1.20,0.46)
	Quantile 4	-10.00(-19.42,-0.58)*	-4.07(-15.64,7.48)	-7.63(-15.27,0.0024)	-2.75(-12.14,6.62)	0.54(-3.47,4.56)	1.47(-2.28 ,5.23)	0.80(-2.16,3.77)	0.93(-1.56,3.44)	-3.00(-11.53,5.52)	0.17(-9.08,9.42)	0.14(-0.56,0.85)	0.11(-0.73,0.94)
	P for trend	0.0109 *	0.22	0.0156 *	0.27	0.86	0.51	0.97	0.55	0.45	0.95	0.41	0.49
Selenium	Continues	-27.72(-54.00, -1.45)*	-26.08(-58.12, 5.96)	-20.87(-42.17, 0.42)	-20.21(-46.22, 5.79)	-3.16(-14.35,8.03)	0.58(-9.82, 11.00)	0.44(-7.83,8.73)	3.35(-3.58,10.28)	-25.46(-49.15, -1.77)*	-23.16(-48.69, 2.37)	1.29(-0.69,3.27)	1.66(-0.67,4.00)
	Quantile 1	Reference	Reference	Reference	Reference	Reference	Reference	Reference	Reference	Reference	Reference	Reference	Reference
	Quantile 2	-5.12(-14.63,4.38)	-4.41(-15.74,6.90)	-4.07(-11.78,3.63)	-3.83(-13.02,5.35)	-2.00(-6.05,2.03)	-1.03(-4.70,2.63)	-0.96(-3.96,2.03)	0.01(-2.43,2.45)	-7.83(-16.39,0.71)	-6.46(-15.46,2.53)	0.053(-0.66,0.77)	0.18(-0.64,1.01)
	Quantile 3	-4.03(-13.49,5.41)	-6.27(-17.39,4.84)	-2.62(-10.28,5.04)	-4.70(-13.72,4.32)	-3.66(-7.68,0.35)	-2.30(-5.91,1.29)	-1.73(-4.71,1.24)	-0.63(-3.04,1.76)	-12.25(-20.75,-3.75)**	-10.37(-19.21,-1.53)*	-0.43(-1.15,0.27)	-0.05(-0.86,0.76)
	Quantile 4	-10.70(-20.20,-1.21)*	-9.05(-20.62,2.51)	-8.21(-15.91,-0.51)*	-7.07(-16.46,2.31)	-1.19(-5.23,2.84)	0.22(-3.52,3.97)	-0.18(-3.17,2.80)	1.21(-1.28,3.71)	-5.42(-13.97,3.11)	-6.64(-15.84,2.55)	0.48(-0.23,1.20)	0.50(-0.34, 1.35)
	P for trend	0.0428 *	0.11	0.061	0.14	0.41	0.90	0.77	0.47	0.12	0.10	0.41	0.36

E2, estradiol; FE2, free estradiol; TT, total testosterone; FT, free testosterone; SHBG, sex hormone-binding globulin; TT/E2, the ratio of TT to E2.

***:p< 0.001; **: p< 0.01, *: p< 0.05

Lead quantile ranges: Q1: -1.66,-0.23; Q2: -0.23,0.18; Q3: 0.18,0.55; Q4: 0.55,2.03.

Mercury quantile ranges: Q1: -1.61,-0.82; Q2: -0.82,-0.30; Q3: -0.30,0.43; Q4: 0.43,3.29.

Cadmium quantile ranges: Q1: -2.65,-1.42; Q2: -1.42,-0.96; Q3: -0.96,-0.41; Q4: -0.41,1.52.

Manganese quantile ranges: Q1: 0.63,2.04; Q2: 2.04,2.26; Q3: 2.26,2.45; Q4: 2.45,3.45.

Selenium quantile ranges: Q1: 4.82,5.18; Q2: 5.18,5.26; Q3: 5.26,5.34; Q4: 5.34,5.85.

Adjusted for age, race, education, marital status, poverty, body mass index, smoking, alcohol assumption, recreational activity, type of menopause, time since menopause, parity, time of blood sampling and energy intake.

After all the potential confounding factors adjusted, this study observed that continuous blood concentration of Pb had significant positive associations with SHBG(β=12.84, 95% CI = (6.77,18.91)) and TT/E2(β=0.69, 95% CI =(0.13,1.25)) and that Cd concentration was significantly positively associated with TT(β=3.25, 95% CI=(1.12,5.38)) and with TT/E2(β=0.76, 95% CI = (0.28,1.24))(all p<0.05). Besides, the corresponding trend tests were also statistically significant.

**Figure 2:**
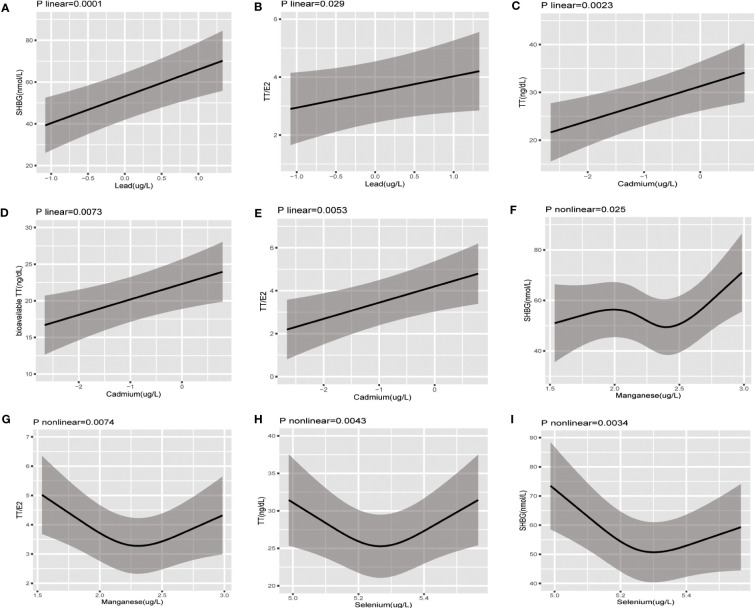
The restricted cubic splines of the association between serum concentration of heavy metal exposure and sex hormones in postmenopausal women; The association was adjusted for age, ethnicity, education level, marital status, poverty-income ratio, body mass index, smoking, alcohol consumption, physical activity, energy intake type of menopause, time since menopause, parity, and time of blood sampling collection. **(A)** Pb-SHBG **(B)** Pb-TT/E2 **(C)** Cd-TT **(D)** Cd-bioavailable TT **(E)** Cd-TT/E2 **(F)** Mn-SHBG **(G)** Mn-SHBG **(H)** Se-TT **(I)**Se-SHBG.

Restricted cubic spline and linear models were adopted to further explore the nonlinear or linear association between blood concentration of heavy metals and sex hormones based on AIC values (**Supplementary Table 1**). As **Figure 2** suggested, Pb concentration had significantly linear negative association with SHBG(p for linear=0.0001) and TT/E2(p for linear=0.029). Cd concentration had significantly linear positive association with TT(p for linear=0.022), fTT(p for linear=0.037), and TT/E2(p for linear=0.0053). However, Mn concentration demonstrated a U-shaped non-linear association with TT/E2(p for non-linear =0.0074) and the inflection point was 2.30. Besides, with the increase of Mn concentration, SHBG increased until Mn concentration reached around 1.98 and decreased afterwards and then increased again around 2.40 (p for non-linear =0.025). Se concentration presented a U-shaped non-linear association with TT(p for non-linear =0.0043) and SHBG(p for non-linear =0.0034) and the inflection points were 5.27 for TT and 5.30 for SHBG. Neither a linear nor nonlinear relationship was noted between other heavy metals and sex hormones.

### 3.3 Subgroup analysis

The adjusted models stratified by type of menopause revealed that in natural menopausal women, continuous blood concentration of Pb had significant positive associations with SHBG(β=9.48, 95%CI=(2.64,16.32)) and TT/E2(β=0.86, 95% CI = (0.19,1.54)) and significant negative association with fE2(β= -6.26, 95% CI=(-12.32,-0.19))(all p<0.05) (**Table 3**). Continuous blood concentration of Cd had significant positive association with TT (β=2.58, 95% CI= (0.12,5.04)) and TT/E2(β=0.93, 95% CI= (0.35,1.51)) (**Table 4**). However, in women who underwent hysterectomy, continuous Pb concentration only had significant positive association with SHBG(β=35.33, 95% CI= (21.00,49.66)) after all covariates adjusted (**Table 3**). No significant associations were noted between Cd concentration and sex hormones in the hysterectomy subgroup (**Table 4**).

**Table 3:** Relationship between blood concentrations of Lead and sex hormones in post-menopausal women stratified by type of menopause(unweighted).

Type of menopause		E2 β(95%CI)		fE2 β(95%CI)		TT β(95%CI)		fTT β(95%CI)		SHBG β(95%CI)		TT/E2 β(95%CI)	
		Crude Model	Adjusted Model	Crude Model	Adjusted Model	Crude Model	Adjusted Model	Crude Model	Adjusted Model	Crude Model	Adjusted Model	Crude Model	Adjusted Model
Natural menopause (n=557)	Continues	-15.29 (-20.57, -10.02) ***	-7.41(-14.92,0.10)	-13.15 (-17.43, -8.87) ***	-6.26(-12.32,-0.19)*	3.62(0.84,6.40)*	1.70(-1.15,4.57)	1.43(-0.66,3.53)	0.31(-1.62,2.24)	15.75(10.12,21.38)***	9.48(2.64,16.32)**	1.77(1.29,2.25)***	0.86(0.19,1.54)*
	Quantile 1	Ref	Ref	Ref	Ref	Ref	Ref	Ref	Ref	Ref	Ref	Ref	Ref
	Quantile 2	-19.70 (-28.56, -10.83) ***	-12.73 (-24.11, -1.35)*	-16.95 (-24.14, -9.75) ***	-11.28 (-20.46, -2.10)*	0.096(-4.57,4.76)	0.48(-3.86,4.82)	-0.23(-3.77,3.29)	0.14(-2.79,3.08)	6.39(-3.14,15.92)	2.32(-8.09,12.74)	1.36(0.55,2.17)***	0.88(-0.13,1.90)
	Quantile 3	-19.57 (-28.32, -10.81) ***	-8.26(-19.80,3.28)	-16.91 (-24.01, -9.80) ***	-7.42(-16.73,1.88)	0.79(-3.81,5.40)	-0.14(-4.54,4.26)	-0.23(-3.72,3.25)	-0.45(-3.43,2.53)	11.60(2.18,21.01)*	4.83(-5.73,15.41)	1.53(0.72,2.33)***	0.28(-0.75,1.31)
	Quantile 4	-24.47 (-33.29, -15.66) ***	-14.25 (-26.35, -2.15)*	-20.97 (-28.13, -13.82) ***	-12.12 (-21.88, -2.36)*	6.67(2.03,11.31)**	3.30(-1.31,7.92)	3.09(-0.42,6.60)	1.01(-2.11,4.13)	23.08(13.60,32.56)***	12.82(1.74,23.90)*	2.85(2.05,3.66)***	1.60(0.52,2.68)**
	P for trend	<0.001***	0.06	<0.001***	0.051	0.0063 **	0.20	0.10	0.62	<0.001***	0.020 *	<0.001***	0.018 *
Hysterectomy (n=136)	Continues	-26.08 (-44.28, -7.88)**	8.23(-19.49,35.95)	-21.94(-36.55,-7.34)**	4.99(-17.71,27.70)	-2.60 (7.19,1.97)	0.52(-4.94,6.00)	-2.62(-5.55,0.30)	-1.29(-4.62,2.04)	21.29(10.46,32.11)***	35.33(21.00,49.66)***	1.38(0.70,2.05)**	-0.19(-1.05,0.66)
	Quantile 1	Ref	Ref	Ref	Ref	Ref	Ref	Ref	Ref	Ref	Ref	Ref	Ref
	Quantile 2	-28.22(-58.06,1.62)	-3.22(-42.91,36.47)	-23.08(-47.03,0.86)	-3.36(-35.84,29.11)	-1.09(-8.66,6.47)	-0.47(-8.28,7.34)	-1.10(-5.94,3.72)	-0.71(-5.45,4.01)	4.39(-13.66,22.45)	5.66(-15.36,26.69)	0.42(-0.70,1.56)	-0.42(-1.63,0.78)
	Quantile 3	-45.67 (-74.84, 16.50)**	0.023(-41.54,41.59)	-37.88 (-61.29, -14.48) **	-2.63(-36.64,31.37)	-4.76(-12.15,2.63)	-1.44(-9.62,6.74)	-3.96(-8.68,0.76)	-2.54(-7.49,2.41)	12.71 (-4.93, 30.37)	23.51(1.49,45.53)*	1.35(0.24,2.46)*	-1.00(-2.27,0.26)
	Quantile 4	-39.98 (-69.36, -10.59) **	-2.06(-44.80,40.66)	-33.41 (-56.99, -9.83)**	-3.61(-38.57,31.34)	-4.97(-12.42,2.47)	-1.35(-9.77,7.05)	-4.55(-9.31,0.21)	-2.97(-8.07,2.11)	30.61(12.83,48.39)***	43.75(21.12,66.39)***	1.90(0.79,3.02)***	-0.28(-1.59,1.01)
	P for trend	0.0038**	0.96	0.0026 **	0.86	0.11	0.71	0.0312 *	0.19	<0.001***	<0.001***	<0.001***	0.55

E2, estradiol; FE2, free estradiol; TT, total testosterone; FT, free testosterone; SHBG, sex hormone-binding globulin; TT/E2, the ratio of TT to E2.

***:p< 0.001; **: p< 0.01, *: p< 0.05

Lead quantile ranges: Q1: -1.66,-0.23; Q2: -0.23,0.18; Q3: 0.18,0.55; Q4: 0.55,2.03.

Adjusted for age, race, education, marital status, poverty, body mass index, smoking, alcohol assumption, recreational activity, type of menopause, time since menopause, parity, time of blood sampling and energy intake.

**Table 4:** Relationship Between blood concentrations of cadmium and sex hormones in post-menopausal women stratified by type of menopause(unweighted).

Type of menopause		E2 β(95%CI)		fE2 β(95%CI)		TT β(95%CI)		fTT β(95%CI)		SHBG β(95%CI)		TT/E2 β(95%CI)	
		Crude Model	Adjusted Model	Crude Model	Adjusted Model	Crude Model	Adjusted Model	Crude Model	Adjusted Model	Crude Model	Adjusted Model	Crude Model	Adjusted Model
Natural menopause (n=557)	Continues	-4.24(-8.47,-0.0084)*	-3.15(-9.65,3.35)	-4.35(-7.79,-0.91)*	-3.38 (-8.63, 1.85)	3.33(1.16,5.50)*	2.58(0.12,5.04)*	1.53(-0.11,3.17)	1.50(-0.15,3.17)	10.77(6.34,15.21)***	5.06(-0.87,11.00)	1.29(0.91, 1.67)***	0.93(0.35,1.51)**
	Quantile 1	Ref	Ref	Ref	Ref	Ref	Ref	Ref	Ref	Ref	Ref	Ref	Ref
	Quantile 2	-14.25 (-23.35, -5.16)**	-5.82(-17.42, 5.77)	-12.63(-20.01,-5.24)***	-5.95 (-15.31, 3.39)	4.29(-0.42,9.00)	-0.49(-4.88,3.89)	2.80(-0.75,6.37)	-0.48(-3.45,2.48)	9.90(0.28,19.52)*	5.76(-4.84,16.38)	1.14(0.32,1.96)**	0.34(-0.68,1.38)
	Quantile 3	-15.43 (-24.17, -6.68)***	-7.16(-18.65,4.31)	-13.87 (-20.97, -6.76)***	-6.85 (-16.11, 2.40)	2.89(-1.64,7.42)	0.099(-4.24,4.44)	0.79(-2.63,4.21)	0.082(-2.85,3.01)	17.95(8.70,27.20)***	3.43(-7.07,13.93)	1.88(1.09,2.67)***	0.81(-0.21,1.83)
	Quantile 4	-8.55(-17.42,0.31)	-5.55(-18.85,7.74)	-8.52(-15.73,-1.32)*	-5.53 (-16.26, 5.18)	6.70(2.11,11.30)**	4.51(-0.51,9.54)	3.29(-0.17,6.77)	2.70(-0.69,6.09)	20.48(11.09,29.87)***	7.48(-4.68,19.64)	2.50(1.70,3.30)***	1.63(0.44,2.82)**
	P for trend	0.044 *	0.34	0.0145 *	0.25	0.011 *	0.12	0.15	0.16	<0.001***	0.30	<0.001***	0.0068 **
Hysterectomy (n=136)	Continues	-10.68 (-23.88, 2.52)	-6.13(-28.60,16.34)	-9.68(-20.28,0.91)	-5.75(-24.13,12.63)	1.80(-1.45, 5.06)	3.70(-0.65,8.06)	0.60(-1.50,2.71)	1.54(-1.14,4.23)	12.18(4.33,20.03)**	10.72(-2.33,23.78)	0.87(0.38,1.36)***	0.043(-0.65,0.74)
	Quantile 1	Ref	Ref	Ref	Ref	Ref	Ref	Ref	Ref	Ref	Ref	Ref	Ref
	Quantile 2	6.96(-23.96,37.89)	-3.65(-41.02,33.71)	5.72(-19.07,30.51)	-2.75(-33.31,27.80)	0.82(-6.85,8.50)	2.82(-4.33,9.97)	0.46(-4.49,5.42)	2.00(-2.37,6.37)	2.26(-15.68,20.20)	-0.26(-21.28,20.75)	0.97(-0.17,2.12)	0.55(-0.60,1.71)
	Quantile 3	-13.34 (-43.11, 16.41)	-11.78(-49.58,26.02)	-13.00 (-36.86, 10.86)	-10.95(-41.87,19.95)	2.47(-4.91,9.86)	7.36(0.12,14.60)*	1.31(-3.46,6.08)	5.07(0.65,9.49)*	10.11(-7.15, 27.38)	-4.26(-25.52,17.00)	0.88(-0.21,1.99)	0.10(-1.06,1.27)
	Quantile 4	-20.22 (-50.41, 9.97)	-0.54(-44.65,43.57)	-18.38 (-42.58, 5.82)	-2.00(-38.08,34.07)	3.44(-4.05,10.93)	7.48(-0.96,15.93)	0.32(-4.51,5.17)	2.73(-2.42,7.90)	33.66(16.14,51.18)***	26.65(1.83,51.46)*	2.07(0.95,3.19)***	0.068(-1.29,1.43)
	P for trend	0.09	0.82	0.061	0.74	0.31	0.040*	0.81	0.12	<0.001***	0.12	<0.001***	0.98

E2, estradiol; FE2, free estradiol; TT, total testosterone; FT, free testosterone; SHBG, sex hormone-binding globulin; TT/E2, the ratio of TT to E2.

***:p< 0.001; **: p< 0.01, *: p< 0.05

Cadmium quantile ranges: Q1: -2.65,-1.42; Q2: -1.42,-0.96; Q3: -0.96,-0.41; Q4: -0.41,1.52.

Adjusted for age, race, education, marital status, poverty, body mass index, smoking, alcohol assumption, recreational activity, type of menopause, time since menopause, parity, time of blood sampling and energy intake.

As for the subgroup analysis stratified by BMI, adjusted models demonstrated that continuous Pb concentration had significantly positive association with SHBG mainly in overweight(β=23.22, 95%CI=(12.18,34.27)) and obese(β=14.61, 95%CI=(7.20,22.01)) postmenopausal women and with TT/E2(β=1.62, 95%CI=(0.17,3.08)) in normal BMI postmenopausal women (**Table 5**). Continuous Cd concentration had significantly negative association with E2 (β= -9.88, 95%CI=(-19.48,-0.29)) and fE2(β=-8.87, 95%CI= (-16.98,-0.75)) mainly in overweight postmenopausal women and had significantly positive association with TT(β=6.48, 95CI%=(3.12,9.83)), fTT(β=4.07, 95%CI=(1.79,6.35)), and TT/E2(β=1.21, 95%CI=(0.56,1.86)) mostly in obese postmenopausal women (**Table 6**).

**Table 5:** Relationship between blood concentrations of lead and sex hormones in post-menopausal women stratified by BMI (unweighted).

BMI		E2 β(95%CI)		fE2 β(95%CI)		TT β(95%CI)		fTT β(95%CI)		SHBG β(95%CI)		TT/E2 β(95%CI)	
<25kg/m2 (n=177)		Crude Model	Adjusted Model	Crude Model	Adjusted Model	Crude Model	Adjusted Model	Crude Model	Adjusted Model	Crude Model	Adjusted Model	Crude Model	Adjusted Model
	Continues	-27.06(-41.23,-12.90)***	-4.58(-27.95,18.78)	-22.27(-33.52,-11.03)***	-4.03(-22.59,14.52)	3.53(-2.56,9.64)	1.31(-3.27,5.89)	1.79(-3.19,6.78)	0.079(-2.53,2.69)	14.52(3.71,25.34)**	14.38(-2.03,30.79)	2.58(1.66,3.50)***	1.62(0.17,3.08)*
	Quantile 1	Ref	Ref	Ref	Ref	Ref	Ref	Ref	Ref	Ref	Ref	Ref	Ref
	Quantile 2	-34.84(-60.57,-9.12)**	-24.10(-62.64,14.42)	-29.74(-50.14,-9.35)**	-21.49(-52.05, 9.05)	-2.64(-13.63,8.34)	-3.12(-10.58,4.33)	-2.82 (-11.81,6.16)	-3.05(-7.27,1.17)	17.31(-2.24, 36.86)	18.67(-8.16,45.51)	1.35(-0.32,3.02)	0.73(-1.66,3.13)
	Quantile 3	-34.48(-60.20,-8.75)**	-16.62(-56.30, 23.05)	-28.91(-49.32,-8.52)**	-14.64(-46.10, 16.81)	0.38(-10.60,11.37)	-1.89(-9.57,5.77)	-1.16 (-10.15, 7.82)	-2.63(-6.98,1.71)	19.58(0.03,39.14)*	23.28(-4.35,50.91)	2.59(0.93,4.27)**	1.30(-1.17,3.77)
	Quantile 4	-47.13(-72.71,-21.55)***	-12.27(-52.84,28.29)	-38.73(-59.02,-18.45)***	-11.11(-43.28, 21.04)	8.54(-2.38,19.47)	3.64(-4.20,11.49)	5.17(-3.76,14.10)	0.52(-3.92,4.97)	29.10(9.66,48.55)**	35.34(7.09,63.59)*	4.44(2.78,6.10)***	3.01(0.48,5.54)*
	P for trend	<0.001***	0.82	<0.001***	0.79	0.10	0.19	0.22	0.49	0.0044 **	0.018 *	<0.001***	0.013 *
25~29.9kg/m2 (n=186)	Continues	-14.25(-24.53,-3.99)**	-0.54(-13.48,12.39)	-11.98(-20.45,-3.52)**	-0.90(-11.87,10.06)	2.37(-2.18,6.93)	-0.57(-5.80,4.66)	0.40(-2.51,3.33)	-2.58(-6.28,1.12)	13.96(3.39,24.53)**	23.22(12.18,34.27)***	1.03(0.23,1.84)*	-0.20(-1.30,0.89)
	Quantile 1	Ref	Ref	Ref	Ref	Ref	Ref	Ref	Ref	Ref	Ref	Ref	Ref
	Quantile 2	-10.51(-25.90,4.87)	1.44(-18.65,15.76)	-9.12(-21.81,3.56)	-1.37(-15.95,13.20)	0.69(-6.07,7.46)	3.20(-3.67,10.08)	0.56(-3.78,4.91)	1.63(-3.23,6.51)	4.30(-11.61, 20.23)	6.57(-8.47,21.61)	0.86(-0.34,2.07)	0.31(-1.14,1.75)
	Quantile 3	-11.38(-26.85,4.08)	-1.10(-17.93,15.71)	-8.75(-21.51,3.99)	-2.70(-14.52,13.98)	-0.82(-7.62,5.98)	-3.03(-9.76,3.68)	-0.87 (-5.25, 3.49)	-3.62(-8.39,1.13)	4.71(-11.29, 20.71)	14.04(-0.66,28.75)	0.97(-0.24,2.19)	-0.59(-2.01,0.82)
	Quantile 4	-19.30(-34.69,-3.92)*	-7.68(-26.13,10.76)	-16.21(-28.90,-3.53)*	-6.75(-22.38,8.87)	4.45(-2.31,11.22)	1.33(-6.04,8.70)	1.49(-2.85,5.84)	-1.88(-7.10,3.34)	14.52(-1.39,30.44)	26.75(10.63,42.88)**	1.36(0.16,2.57)*	-0.08(-1.64,1.47)
	P for trend	0.017*	0.46	0.018 *	0.46	0.27	0.79	0.65	0.17	0.084	0.001 **	0.0308 *	0.61
≥30kg/m2 (n=328)	Continues	-14.58(-21.25,-7.91)***	-0.88(-9.42,7.66)	-12.81(-18.30,-7.33)***	-1.62(-8.75, 5.51)	2.15(-0.82,5.12)	3.33(-0.62,7.29)	0.72(-1.28, 2.73)	1.46(-1.23, 4.15)	11.41(5.70,17.13)***	14.61(7.20,22.01)***	0.98(0.47,1.50)***	0.68(-0.08, 1.44)
	Quantile 1	Ref	Ref	Ref	Ref	Ref	Ref	Ref	Ref	Ref	Ref	Ref	Ref
	Quantile 2	-19.23(-30.02,-8.45)***	-5.76(-18.54,7.01)	-16.32(-25.18,-7.48)***	-6.09(-16.73,4.55)	-3.57(-8.41,1.26)	-3.56(-9.43, 2.30)	-2.58 (-5.86, 0.68)	-2.67(-6.65, 1.32)	2.22(-7.15,11.59)	4.98(-6.17,16.14)	0.65(-0.19,1.49)	0.42(-0.72, 1.56)
	Quantile 3	-24.54(-35.43,-13.66)***	-7.17(-20.50,6.16)	-21.55(-30.48,-12.62)***	-7.77(-18.88,3.33)	1.35(-3.52, 6.24)	2.58(-3.54, 8.70)	0.55(-2.74, 3.86)	1.44(-2.72,5.60)	8.08(-1.37,17.54)	9.01(-2.63,20.65)	0.76(-0.09, 1.61)	0.30(-0.89, 1.48)
	Quantile 4	-25.91(-36.70,-15.13)***	-4.34(-18.55, 9.87)	-22.45(-31.30,-13.61)***	-5.05(-16.89,6.79)	1.97(-2.86,6.81)	3.88(-2.65,10.40)	0.25(-3.01, 3.52)	1.55(-2.89,5.98)	17.00(7.63,26.37)***	21.05(8.64,33.46)***	1.75(0.91,2.59)***	1.42(0.15, 2.68) ***
	P for trend	<0.001***	0.57	<0.001***	0.42	0.16	0.064	0.45	0.16	<0.001***	<0.001***	<0.001***	0.042 *

E2, estradiol; FE2, free estradiol; TT, total testosterone; FT, free testosterone; SHBG, sex hormone-binding globulin; TT/E2, the ratio of TT to E2.

Lead quantile ranges: Q1: -1.66,-0.23; Q2: -0.23,0.18; Q3: 0.18,0.55; Q4: 0.55,2.03.

Adjusted for age, race, education, marital status, poverty, body mass index, smoking, alcohol assumption, recreational activity, type of menopause, time since menopause, parity, time of blood sampling and energy intake.

**Table 6:** Relationship between blood concentrations of cadmium and sex hormones in post-menopausal women stratified by BMI (unweighted).

BMI		E2 β(95%CI)		fE2 β(95%CI)		TT β(95%CI)		fTT β(95%CI)		SHBG β(95%CI)		TT/E2 β(95%CI)	
<25kg/m2 (n=177)		Crude Model	Adjusted Model	Crude Model	Adjusted Model	Crude Model	Adjusted Model	Crude Model	Adjusted Model	Crude Model	Adjusted Model	Crude Model	Adjusted Model
	Continues	-5.88(-18.13,6.35)	-0.77(-22.52,20.98)	-5.60(-15.34,4.12)	-1.27(-18.55,16.00)	2.33(-2.75,7.42)	1.69(-2.56, 5.95)	0.25(-3.90,4.41)	0.46(-1.95,2.89)	15.63(6.74,24.52)***	12.33(-2.96,27.63)	1.38(0.57,2.18)***	0.71(-0.66,2.10)
	Quantile 1	Ref	Ref	Ref	Ref	Ref	Ref	Ref	Ref	Ref	Ref	Ref	Ref
	Quantile 2	-33.60(-59.93,-7.28)*	-22.84(-62.64,16.96)	-27.90(-48.80,-6.99)**	-19.71(-51.29,11.86)	7.21(-3.91,18.34)	-1.40(-9.18,6.37)	4.76(-4.32,13.86)	-1.44(-5.88,2.99)	27.51(8.27,46.76)**	15.58(-12.46,43.64)	2.47(0.73,4.21)**	1.09(-1.41,3.60)
	Quantile 3	-28.33(-54.22,-2.45)*	-14.39(-55.09,26.29)	-24.38(-44.94,-3.82)*	-13.57(-45.86,18.70)	3.15(-7.78,14.09)	1.69(-6.25,9.64)	0.44(-8.50,9.38)	0.24(-4.29,4.78)	28.22(9.30,47.15)**	20.58(-8.09,49.26)	2.14(0.43,3.85)*	0.43(-2.12,3.01)
	Quantile 4	-10.97(-37.14,15.20)	-7.38(-51.16,36.39)	-10.41(-31.19, 10.37)	-7.28(-42.01,27.44)	6.62(-4.43,17.69)	4.63(-3.91,13.19)	1.89(-7.14,10.93)	1.31(-3.56,6.20)	38.15(19.02,57.29)***	29.00(-1.85,59.86)	3.40(1.67,5.13)***	2.29(-0.46,5.05)
	P for trend	0.50	0.89	0.40	0.83	0.37	0.21	0.92	0.46	<0.001***	0.073	<0.001***	0.14
25~29.9kg/m2 (n=186)	Continues	-7.57(-15.05,-0.083)*	-9.88(-19.48,-0.29)*	-6.89(-13.05,-0.73)*	-8.87(-16.98,-0.75)*	3.33(0.072,6.59)*	-0.67(-4.62,3.26)	1.66(-0.43,3.76)	-0.51(-3.32,2.30)	7.62(-0.073,15.31)	1.45(-7.48,10.38)	0.82(0.24,1.40)**	0.10(-0.72,0.92)
	Quantile 1	Ref	Ref	Ref	Ref	Ref	Ref	Ref	Ref	Ref	Ref	Ref	Ref
	Quantile 2	-9.19(-24.41,6.02)	-11.66(-27.94,4.60)	-8.48(-21.01,4.04)	-10.67(-24.44,3.09)	1.05(-5.58,7.68)	-5.06(-11.66,1.53)	0.14(-4.11,4.40)	-4.11(-8.79,0.56)	8.05(-7.53,23.64)	7.24(-7.75,22.23)	0.10(-1.07,1.29)	-0.62(-2.01,0.76)
	Quantile 3	-9.07(-25.19,7.05)	-12.66(-30.64,5.31)	-8.87(-22.15,4.39)	-11.27(-26.48,3.94)	4.43(-2.60,11.46)	-1.55(-8.84,5.73)	1.61(-2.90,6.12)	-0.66(-5.83,4.50)	16.96(0.44,33.48)*	-1.26(-17.83,15.31)	0.66(-0.59,1.91)	-0.47(-2.00,1.06)
	Quantile 4	-14.11(-30.05,1.83)	-15.01(-34.86,4.82)	-12.85(-25.97,0.27)	-13.46(-30.25,3.33)	4.73(-2.21,11.69)	-3.44(-11.49,4.59)	2.48(-1.97,6.94)	-2.75(-8.46, 2.95)	10.51(-5.81,26.85)	3.39(-14.89,21.68)	1.06(-0.18,2.30)	-0.52(-2.21,1.16)
	P for trend	0.10	0.14	0.069	0.12	0.11	0.62	0.20	0.64	0.13	0.99	0.056	0.59
≥30kg/m2 (n=328)	Continues	-6.93(-12.08,-1.78)**	-2.81(-10.24,4.60)	-6.57(-10.80,-2.33)*	-3.16(-9.35,3.02)	3.71(1.48, 5.93)**	6.48(3.12,9.83)***	2.41(0.91,3.91)**	4.07(1.79,6.35)***	3.53(-0.88,7.95)	6.66(0.063,13.26)*	0.99(0.60,1.37)***	1.21(0.56,1.86)***
	Quantile 1	Ref	Ref	Ref	Ref	Ref	Ref	Ref	Ref	Ref	Ref	Ref	Ref
	Quantile 2	-1.27(-12.37,9.81)	2.10(-10.67,14.87)	-2.00(-11.11,7.10)	0.54(-10.11,11.20)	1.41(-3.39,6.22)	1.79(-4.00,7.60)	0.68(-2.55,3.92)	0.78(-3.15,4.72)	2.34(-7.18,11.88)	3.50(-7.91,14.93)	0.31(-0.52,1.14)	-0.21(-1.34,0.90)
	Quantile 3	-13.64(-25.05,-2.24)*	-5.48(-18.87,7.89)	-12.91(-22.29,-3.54)**	-5.54(-16.71,5.62)	4.37(-0.57,9.31)	6.01(-0.071,12.09)	2.46(-0.86,5.80)	3.96(-0.16,8.10)	9.38(-0.42,19.18)	2.80(-9.16,14.78)	1.64(0.78,2.49)***	1.15(-0.027,2.33)
	Quantile 4	-12.41(-23.75,-1.07)*	-2.68(-17.62,12.26)	-11.69(-21.01,-2.38)*	-3.46(-15.93,9.00)	7.28(2.37,12.19)***	11.18(4.39,17.97)**	4.78(1.47, 8.10)**	6.99(2.38,11.60)**	4.92(-4.81,14.67)	9.76(-3.60,23.12)	1.65(0.80,2.50)***	1.49(0.17,2.81)*
	P for trend	0.0062**	0.48	0.0021**	0.38	0.0015**	<0.001***	0.0022 **	0.0013 **	0.16	0.20	<0.001***	0.0057**

E2, estradiol; FE2, free estradiol; TT, total testosterone; FT, free testosterone; SHBG, sex hormone-binding globulin; TT/E2, the ratio of TT to E2.

Cadmium quantile ranges: Q1: -2.65,-1.42; Q2: -1.42,-0.96; Q3: -0.96,-0.41; Q4: -0.41,1.52.

Adjusted for age, race, education, marital status, poverty, body mass index, smoking, alcohol assumption, recreational activity, type of menopause, time since menopause, parity, time of blood sampling and energy intake.

Restricted cubic spline and linear models were also adopted to further explore the nonlinear or linear association in subgroup based on AIC values (**Supplementary Table 2**). As for women who underwent natural menopause, the associations remained consistent with the aforementioned descriptions, except the association between Se concentration and TT(p linear=0.78), which was statistically significant in women who underwent hysterectomy(p non-linear=0.0001), though. In terms of women who underwent hysterectomy, we only observed the positive association of Pb concentration with SHBG (p linear= 0.0001) and U-shaped association of Se concentration with TT (p non-linear= 0.0001) and SHBG (p non-linear=0.025) (**Supplementary Figures 1, 2**).

When stratified by BMI, the curves describing the linear and nonlinear association differed in various BMI groups (**Supplementary Table 2**, **Supplementary Figures 3-5**), after all the potential confounding factors adjusted. In postmenopausal women whose BMI was less than <25kg/m2, we observed significant linear association between Pb concentration and TT/E2 (p linear=0.031) and non-linear association between Mn concentration and TT/E2 (p non-linear=0.018). In overweight (BMI:25~29.9kg/m2) postmenopausal women, we only detected the significant linear association between Pb concentration and SHBG(p linear<0.0001) and non-linear association of Se concentration with TT(p non-linear=0.0025) and SHBG(p non-linear=0.032). In obese(BMI>30 kg/m2) postmenopausal women, the associations remained consistent with the aforementioned descriptions in overall population, except the association between the Mn concentration and TT/E2(p linear=0.13).

### 3.4 Sensitivity analysis

The association between blood concentration of heavy metals and sex hormones didn’t present substantial change when the NHANES sampling weight was adopted, whether in overall population (**Supplementary Table 3**) or in subgroup analysis (**Supplementary Tables 4-7**). When blood concentration of heavy metals served as continuous variables in adjusted models, Cd concentration appeared new significant positive association with SHBG in overweight and obese postmenopausal women, other associations changed little (**Supplementary Table 5**).

## 4 Discussion

To the extent of our knowledge, our study is the first study to explore the association between heavy metal exposure and sex hormones altogether in 614 postmenopausal women based on the data extracted from 2013-2014 and 2015-2016 year cycles of NHANES. In overall population, Cd concentration had linear positive association with TT, bioavailable TT and TT/E2, which was more apparent in natural menopausal and obese women. Pb concentration had linear positive association with SHBG, which was apparent in nearly all subgroups except in normal BMI group, and TT/E2, which was apparent in natural menopausal and normal BMI women. Mn concentration had non-linear association with SHBG, which was more apparent in natural menopausal and obese women, and TT/E2, which was more apparent in natural menopausal and normal BMI women. Se concentration also had non-linear association with TT, which was more apparent in hysterectomy, overweight and obese women, and SHBG, which was apparent in nearly all subgroups except in normal BMI group.

### 4.1 The influence of cadmium on sex hormones

The major source of exposure to Cd is inhalation of cigarette smoke, whereas among non-smokers, consumption of contaminated foods, such as shellfish, rice, grains, and vegetables, is the main source of exposure (35). As endocrine disrupting chemical, Cd has been shown to affect the hypothalamic-pituitary-gonadal (HPG) axis (36–38)and thus would disrupt the estrogen-androgen balance. However, the evidence is equivocal. Kim’s study (39) suggested a potential association between increasing levels of Cd with higher TT, anti-Müllerian hormone(AMH), and SHBG among healthy women of reproductive age, which were mainly non-smokers. Ali’s study (40) indicated a significant positive association between blood concentration of Cd and serum TT levels, as well as a significant inverse association between Cd and serum E2 levels in 438 Swedish postmenopausal women without hormone replacement therapy. However, subgroup analysis stratified by type of menopause and BMI in postmenopausal women and the detailed trends have not been clarified in the aforementioned studies.

Our study involved smoking status and other lifestyles as covariates to further assess the role of Cd that it played in the alteration of sex hormones among postmenopausal women, considering smoking as one of the primary sources of Cd exposure. Multivariate-adjusted linear models showed Cd concentration could serve as an independent risk factor to increase TT(β:3.25, 95% CI: (1.12, 5.38)), bioavailable TT(β:1.78, 95% CI: (0.36,3.21)) and TT/E2(β:0.76, 95% CI: (0.28,1.24) and the influence was more apparent in obese and natural menopausal women. The main conclusion was consistent with Kim’s and Ali’s study, but the deleterious effect of Cd exposure on estrogen level was only observed in the unadjusted models. The mechanism that Cd could stimulate the increase of TT remains elusive. On one hand, Cd has been reported to inhibit luteinizing hormone (LH)-induced ovarian aromatase activity (conversion of testosterone to 17β-estradiol) and P450 aroma gene expression *in vitro* (41). We then assessed the testosterone/estradiol ratio towards the blood concentration of Cd to indirectly elucidate this aspect of LH-induced aromatase activity in our study population. Interestingly, we noted a significant positive association between Cd concentration and TT/E2, which indicated that Cd might interfere with the LH-induced P450 aromatase activity to further disturb the estrogen–androgen balance; however, further investigations are warranted. On the other hand, increased SHBG may be associated with the increase in TT. We did notice a significant positive association of Cd concentration with SHBG but the association was non-significant after adjusting all the covariates, though.

In terms of stratified analysis, for women who underwent hysterectomy, the effect of Cd on increasing TT was not statistically significant. It is speculated that the reduced blood supply of ovaries due to the removal of uterus lead to diminished ovarian function, which further eliminates the estrogen-testosterone interplay, potentially reducing the impact of cadmium on testosterone levels. Besides, for obese individuals, adipose tissue (fat cells) might serve as a reservoir for cadmium accumulation in the body (42), which can subsequently be released into the bloodstream and influence testosterone levels. Non-obese women without hysterectomy may have lower cadmium accumulation in adipose tissue, leading to a reduced effect of Cd on testosterone levels. Therefore, we could speculate that accumulated Cd stored in adipose tissue mainly interferes with aromatization to disturb the estrogen–androgen balance, especially in obese natural menopause women.

### 4.2 The influence of Pb on sex hormones

Pb is pervasive in our daily life, in the form of soil, drinking water and other consumer products. Our study revealed that Pb exposure had linear positive association with SHBG in the adjusted models, which was apparent in nearly all subgroups except in normal BMI group, thus influencing the utility of SHBG as a sensitive biomarker of the degree of inflammation in metabolic diseases (8). As SHBG levels are also effected by factors related to metabolism, such as insulin resistance and lipid profiles, which can be influenced by both lead exposure (43, 44) and BMI. Therefore, in the normal BMI group, where metabolic dysregulation may be less prevalent, the impact of Pb exposure on SHBG levels may be attenuated.

However, the effect of Pb exposure on reducing estrogen level was only observed in natural menopausal women other than overall population in the adjusted models, in comparison with previous research findings (45). He et al. (46) reported that Pb might cause ovarian malfunction by inducing ovary microstructural damages, including granulosa cells disorganization, follicle atresia and interstitial cell degeneration, and thus disrupting ovarian steroidogenesis. It is assumed that in hysterectomy women, who experience diminished ovarian function and lower estrogen levels due to the reduced blood supply, the additional impact of Pb on estrogen metabolism in ovary weakened or not as apparent.

### 4.3 The influence of Mn on sex hormones

Mn and Se are both essential trace nutrients for normal growth and reproduction in all known forms of life, and both inadequate and excessive exposure can cause several serious health outcomes (47). Mn is contained in groundwater and soil at low levels and people are frequently exposed *via* drinking water, air, soil, and food (48). Most of the studies focused on its association with sex hormones in males, as seen in the inverse monotonic association of Mn with SHBG in fertile men (49) and with TT in male workers (50). However, in older men aged 50–75 years, positive association was detected between the concentration of SHBG-Mn (51). Few literature has focused on the association between Mn and SHBG in postmenopausal women.

This study observed the non-linear association between Mn and SHBG, which was more pronounced in natural menopausal and obese women. SHBG increased gradually as Mn concentration increased after reaching a certain threshold concentration. Besides, Mn concentration presented a U-shaped association with TT/E2, which was more apparent in natural menopausal and normal BMI women. Therefore, it is speculated that various degree of Mn exposure could be complicated in the regulation of LH-induced P450 aromatase activity and SHBG levels to further disturb the estrogen–androgen balance in ovary. However, the association between Mn exposure and TT or E2 was not statistically significant when adjusted for all the covariates. In words, small degree of Mn exposure would have little influence on SHBG, but excessive dosage was related to high level of SHBG. The findings have potential clinical implications, as they highlight the complex relationship between Mn exposure, SHBG levels, and hormonal balance. Understanding these associations may help in identifying potential health risks associated with Mn exposure and provide insights into the effects of hormonal changes, such as natural menopause and obesity, on hormone regulation. The mechanism underlying the influence of Mn concentration on SHBG and TT/E2 remains unclear, which points new direction for the current research.

### 4.4 The influence of Se on sex hormones

Se functions as an antioxidant as well as a peroxynitrite scavenger. It is the primary component of glutathione peroxidase, which reduces free radical production and lipoprotein peroxidation (52). Many biochemical studies have provided the strong rationales for the concept that Se status also interacts with sex hormones secretion (14). Xiao’ s (53) study has showed that Se was positively associated with TT in children and adolescents. Coskun et al. (54) reported plasma concentrations of Se had a negative association with LH, total testosterone (tT) in women with polycystic ovary syndrome, for whom selenium supplementation could significantly reduce tT (55). However, for now, no study has focused on the investigation into the association between Se concentration and sex hormones in postmenopausal women.

In contrast to the traditional belief that Se could boost levels of testosterone in men (56), our study observed that Se had U-shaped non-linear association with TT, which was more apparent in overweight, obese and hysterectomy women, and with SHBG, which was apparent in nearly all subgroups except in normal BMI group. The U-shaped association suggests that both insufficient and excessive Se levels may impact testosterone levels. In overweight, obese, and hysterectomy women, hormonal imbalances and altered metabolic profiles could contribute to the non-linear relationship between Se and SHBG, which in turn influences TT level. The result indicated that in postmenopausal women adequate amount of Se intake would decrease TT and SHBG, while excess intake would increase them, which provides theoretic foundation for the appropriate dosage of Se supplementation for body.

This study has several strengths. First, our sample involved 614 postmenopausal women, the size of which was larger and nationally representative. Second, the study adjusted for richer covariate data than previous observational studies and this study includes abundant demographic characteristics, lifestyles, energy intake and time of blood collection as covariates, which renders our results more realistic. Third, restricted cubic spline plots depicted a more accurate association between the concentration of heavy metals and sex hormones, which made up for the deficiency of previous studies, and stratified analysis elucidated in which group the effect was more pronounced. Last but not least, sampling weights were adopted to reanalyze the dataset as sensitivity analysis to provide more robustness for our main conclusions.

However, some limitations exist in this study. First, this study was cross-sectional and hence causality cannot be concluded. Second, although a broad array of covariates has been adopted in the regression model, other potential confounding variates may also serve as contributing factors, such as some endocrine disease, the variety and dosage of medications. Third, in this study, no details regarding the mechanism behind the influence of heavy metals on hypothalamus-pituitary gland-gonads axis were revealed. Further verification on the link between Cd, Pb, Mn and Se concentration and sex hormones is of great demand.

In summary, our study highlighted the association between heavy metals and sex hormones in light of the possible implication of Cd, Pb, Mn and Se into disruption of sex hormone levels, which would offer more precise evaluation and understanding of sex hormone alterations in postmenopausal women. Although this research does not show that heavy metals are a direct cause of sex hormone fluctuations because observed associations may have other explanations; the findings show the importance of more knowledge about how environmental contaminants may impact sex hormones. Therefore, it is of vital importance to underline the need to mitigate exposure to the ubiquitous environmental contaminant of heavy metals through modifiable lifestyle changes for women’s reproductive health.

## Data availability statement

The original contributions presented in the study are included in the article/**Supplementary Material**. Further inquiries can be directed to the corresponding author.

## Ethics statement

The studies involving human participants were reviewed and approved by NCHS Ethics Review Board (ERB) Approval. The patients/participants provided their written informed consent to participate in this study.

## Author contributions

Conceptualization, formal analysis, methodology and software: WZ. Supervision: YC, JL. Writing – original draft: WZ. Writing – review & editing:YC, JL. All authors contributed to the article and approved the submitted version.
