# Supplementary material for: The association between blood heavy metals level and sex hormones among postmenopausal women in the US
Source: Front Endocrinol (Lausanne). 2023 Jul 18;14:1175011. doi: 10.3389/fendo.2023.1175011 (PMC10391169; doi:10.3389/fendo.2023.1175011)
Supplement: Supplementary file 1 [file DataSheet_1.docx]

Supplementary Material

# Supplementary Tables

| Metals  Sex hormones | lead | | mercury | | cadmium | | manganese | | selenium | |
| --- | --- | --- | --- | --- | --- | --- | --- | --- | --- | --- |
|  | linear | RCS | linear | RCS | linear | RCS | linear | RCS | linear | RCS |
| E2 | - | - | - | - | - | - | - | - | - | - |
| fE2 | - | - | - | - | - | - | -- | - | - | - |
| TT | - | - | - | - | **3627.36** | 3628.30 | - | - | 3636.94 | **3631.41** |
| fT | - | - | - | - | **3262.61** | 3263.72 | - | - | - | - |
| SHBG | **4407.94** | 4408.88 | - | - | **-** | - | 4421.60 | **4416.91** | 4419.26 | **4413.62** |
| TT/E2 | **2326.52** | 2329.31 | - | - | **2321.39** | 2322.76 | 2328.72 | **2323.55** | - | - |

**S Table 1**. Comparison of AIC values for the linear and Restricted cubic splines models of association between blood concentration of heavy metals and sex hormones

All models were adjusted for age, race, education, marital status, poverty, body mass index, smoking, alcohol assumption, recreational activity, type of menopause, time since menopause, parity, time of blood sampling and energy intake. The numbers in bold letters indicate that the AIC was considered smaller.

**S Table 2.** Comparison of AIC values for the linear and nonlinear models in subgroup analyses of association between blood concentration of heavy metals and sex hormones

The numbers in bold letters indicate that the AIC was considered smaller.

**Hysterectomy****†**

| Metals  Sex hormones | lead | | mercury | | cadmium | | manganese | | selenium | |
| --- | --- | --- | --- | --- | --- | --- | --- | --- | --- | --- |
|  | linear | RCS | linear | RCS | linear | RCS | linear | RCS | linear | RCS |
| E2 | - | - | - | - | - | - | - | - | - | - |
| fE2 | - | - | - | - | - | - | - | - | - | - |
| TT | - | - | - | - | **777.63** | 780.74 | - | - | 780.35 | **762.70** |
| fT | - | - | - | - | **683.32** | 685.73 | - | - | - | - |
| SHBG | **968.004** | 969.72 | - | - | **-** | - | **994.57** | 998.69 | 995.03 | **991.47** |
| TT/E2 | **418.82** | 420.14 | - | - | **419.05** | 424.87 | **418.70** | 422.46 | - | - |

All models were adjusted for age, race, education, marital status, poverty, body mass index, smoking, alcohol assumption, recreational activity, time since menopause, parity, time of blood sampling and energy intake.

| Metals  Sex hormones | lead | | mercury | | cadmium | | manganese | | selenium | |
| --- | --- | --- | --- | --- | --- | --- | --- | --- | --- | --- |
|  | linear | RCS | linear | RCS | linear | RCS | linear | RCS | linear | RCS |
| E2 | - | - | - | - | - | - | - | - | - | - |
| fE2 | **3881.37** | 3886.41 | - | - | - | - | - | - | - | - |
| TT | - | - | - | - | **3281.22** | 3284.99 | - | - | **3285.61** | 3286.34 |
| fT | - | - | - | - | **2969.40** | 2973.31 | - | - | - | - |
| SHBG | **3977.57** | 3980.34 | - | - | **-** | - | 3981.87 | **3978.04** | 3982.16 | **3979.05** |
| TT/E2 | **2133.23** | 2136.81 | - | - | **2129.41** | 2134.27 | 2138.97 | **2117.49** | - | - |

**Natural menopause†**

All models were adjusted for age, race, education, marital status, poverty, body mass index, smoking, alcohol assumption, recreational activity, time since menopause, parity, time of blood sampling and energy intake.

| Metals  Sex hormones | lead | | mercury | | cadmium | | manganese | | selenium | |
| --- | --- | --- | --- | --- | --- | --- | --- | --- | --- | --- |
|  | linear | RCS | linear | RCS | linear | RCS | linear | RCS | linear | RCS |
| E2 | - | - | - | - | - | - | - | - | - | - |
| fE2 | - | - | - | - | - | - | -- | - | - | - |
| TT | - | - | - | - | **984.86** | 990.21 | - | - | **985.41** | 990.06 |
| fT | - | - | - | - | **847.59** | 853.19 | - | - | - | - |
| SHBG | **1296.38** | 1300.14 | - | - | - | - | **1296.32** | 1299.23 | **1299.42** | 1300.93 |
| TT/E2 | **705.90** | 711.017 | - | - | **710.28** | 715.91 | 707.19 | **703.13** | - | - |

**BMI<25kg/m^2^&**

All models were adjusted for age, race, education, marital status, poverty, smoking, alcohol assumption, recreational activity, type of menopause, time since menopause, parity, time of blood sampling and energy intake. The numbers in bold letters indicate that the AIC was considered smaller.

| Metals  Sex hormones | lead | | mercury | | cadmium | | manganese | | selenium | |
| --- | --- | --- | --- | --- | --- | --- | --- | --- | --- | --- |
|  | linear | RCS | linear | RCS | linear | RCS | linear | RCS | linear | RCS |
| E2 | - | - | - | - | - | - | - | - | - | - |
| fE2 | - | - | - | - | - | - | - | - | - | - |
| TT | - | - | - | - | **1102.95** | 1108.38 | - | - | 1102.92 | **1092.56** |
| fT | - | - | - | - | **1011.51** | 1016.94 | - | - | - | - |
| SHBG | **1304.88** | 1305.09 | - | - | - | - | **1321.13** | 1321.81 | 1319.46 | **1314.78** |
| TT/E2 | **680.13** | 684.49 | - | - | **680.22** | 684.17 | **679.29** | 680.43 | - | - |

**BMI 25~29.9kg/m^2^&**

| Metals  Sex hormones | lead | | mercury | | cadmium | | manganese | | selenium | |
| --- | --- | --- | --- | --- | --- | --- | --- | --- | --- | --- |
|  | linear | RCS | linear | RCS | linear | RCS | linear | RCS | linear | RCS |
| E2 | - | - | - | - | - | - | - | - | - | - |
| fE2 | - | - | - | - | - | - | - | - | - | - |
| TT | - | - | - | - | **984.86** | 990.21 | - | - | **985.41** | 990.06 |
| fT | - | - | - | - | **847.59** | 853.19 | - | - | - | - |
| SHBG | **1296.38** | 1300.14 | - | - | - | - | **1296.32** | 1299.23 | **1299.42** | 1300.93 |
| TT/E2 | **705.90** | 711.017 | - | - | **710.28** | 715.91 | 707.19 | **703.13** | - | - |

All models were adjusted for age, race, education, marital status, poverty, smoking, alcohol assumption, recreational activity, type of menopause, time since menopause, parity, time of blood sampling and energy intake. The numbers in bold letters indicate that the AIC was considered smaller.

| Metals  Sex hormones | lead | | mercury | | cadmium | | manganese | | selenium | |
| --- | --- | --- | --- | --- | --- | --- | --- | --- | --- | --- |
|  | linear | RCS | linear | RCS | linear | RCS | linear | RCS | linear | RCS |
| E2 | - | - | - | - | - | - | - | - | - | - |
| fE2 | - | - | - | - | - | - | - | - | - | - |
| TT | - | - | - | - | **1990.40** | 1992.22 | - | - | 2005.62 | **2001.45** |
| fT | - | - | - | - | **1806.45** | 1807.09 | - | - | - | - |
| SHBG | **2302.67** | 2306.83 | - | - | - | - | 2318.50 | **2317.33** | 2318.50 | **2317.33** |
| TT/E2 | **1216.68** | 1221.35 | - | - | **1205.75** | 1208.27 | **1217.59** | 1219.98 | - | - |

**BMI≥30kg/m^2^&**

All models were adjusted for age, race, education, marital status, poverty, smoking, alcohol assumption, recreational activity, type of menopause, time since menopause, parity, time of blood sampling and energy intake. The numbers in bold letters indicate that the AIC was considered smaller.

**S Table3.** Relationship between blood concentrations of heavy metals and sex hormones in post-menopausal women（weighted）

| metal |  | E2  β(95%CI) | | fE2  β(95%CI) | | TT  β(95%CI) | | fTT  β(95%CI) | | SHBG  β(95%CI) | | TT/E2  β(95%CI) | |
| --- | --- | --- | --- | --- | --- | --- | --- | --- | --- | --- | --- | --- | --- |
|  |  | Crude Model | Adjusted Model | Crude Model | Adjusted Model | Crude Model | Adjusted Model | Crude Model | Adjusted Model | Crude Model | Adjusted Model | Crude Model | Adjusted Model |
| Lead | Continues | **-21.73 (-28.13, -15.33)***** | -3.37( -9.38, 2.64) | **-18.76(-24.31,-13.20)***** | -3.5(-9.13, 2.12) | 1.37(-0.96,3.70) | 2.46(-0.56, 5.48) | -0.08(-1.69,1.53) | 0.64(-1.65, 2.92) | **16.61(11.97,21.25)***** | **13.7( 6.64, 20.76)**** | **1.78(1.36,2.20)***** | **0.67( 0.04, 1.30)*** |
|  | Q1 | Ref | Ref | Ref | Ref | Ref | Ref | Ref | Ref | Ref | Ref | Ref | Ref |
|  | Q2 | **-32.63(-44.97,-20.29)***** | **-15.67(-29.05,-2.28)*** | **-27.88(-37.92,-17.83) ***** | **14.23(-25.60,-2.86)*** | 0.52(-3.80,4.84) | 2.24(-1.80, 6.28) | 0.09(-2.87,3.06) | 1.38(-1.74, 4.51) | 7.71(-1.95,17.37) | 3.35( -6.75, 13.45) | **1.94(1.10,2.78) ***** | **1( 0.17, 1.83)*** |
|  | Q3 | **-31.76(-44.85,-18.66)***** | -8.01(-24.38, 8.35) | **-27.1(-38.21,-15.98)***** | -7.59(-21.64, 6.45) | -1(-4.67,2.68) | -0.16(-3.45, 3.13) | -1.25(-3.90,1.40) | -0.33(-2.86, 2.20) | **11.03( 0.88,21.17)*** | 3.95( -6.21, 14.11) | **1.84(1.11,2.56) ***** | 0.32(-0.31, 0.95) |
|  | Q4 | **-35.39(-48.05,-22.72)***** | -10.91(-25.53, 3.71) | **-30.47(-40.91,-20.02) ***** | -10.26(-22.28, 1.76) | 3.38(-1.14,7.89) | 5.05(-0.62,10.71) | 0.69(-2.33,3.71) | 1.91(-1.80, 5.62) | **25.97(16.43,35.52)***** | **19.66(7.27, 32.05)*** | **3.1(2.31,3.90)***** | **1.6( 0.52, 2.69)*** |
| Mercury | Continues | -0.52(-4.86,3.82) | -1.19( -6.89, 4.51) | -0.34(-4.05,3.37) | -1.11( -6.06, 3.84) | -1.12(-2.38,0.13) | -0.16(-1.63, 1.32) | -0.87(-1.75,0.02) | -0.13(-1.18, 0.92) | 2.95(-1.91,7.81 | -0.15(-3.80,  3.50) | 0.21(-0.11,  0.53) | 0.11(-0.24, 0.45) |
|  | Q1 | Ref | Ref | Ref | Ref | Ref | Ref | Ref | Ref | Ref | Ref | Ref | Ref |
|  | Q2 | 2.64(-10.82,16.09) | 4.58( -8.58,17.74) | 2.44(-8.77,13.66) | 3.6( -7.40,14.60) | -1.91(-6.00, 2.19) | -1.56( -6.81, 3.69) | -1.14(-3.90, 1.62) | -1.17(-4.62, 2.29) | -4.51(-18.74, 9.71) | -3.35(-18.03, 11.33) | 0.09(-0.62,0.80) | 0.17(-0.73, 1.07) |
|  | Q3 | 9.79( -3.96,23.55) | 10.49( -3.18,24.15) | 7.17(-3.58,17.93) | 6.92( -3.43,17.28) | **-3.17(-5.71,-0.63)*** | -1.14( -5.59, 3.32) | **-2.28(-4.09,-0.47)*** | -1.12(-4.18, 1.94) | 4.36( -8.92,17.64) | 6.96( -8.04, 21.96) | -0.49(-1.19,0.22) | -0.58(-1.36, 0.20) |
|  | Q4 | -1.72(-11.51, 8.08) | -1.44(-14.91,12.04) | -1.12(-9.53, 7.29) | -1.43(-13.06,10.20) | -2.84(-6.25, 0.56) | -0.42( -4.90, 4.06) | -2.1(-4.61, 0.41) | -0.45(-3.80, 2.90) | 5.77( -8.86,20.41) | 1.08( -9.61, 11.76) | 0.52(-0.38,1.43) | 0.21(-0.79, 1.21) |
| cadmium | Continues | **-8.85(-15.32,-2.39)**** | -7.84(-17.20, 1.52) | **-8.38(-13.49,-3.26)**** | -7.56(-15.80, 0.67) | **3.07(1.57,4.57)***** | **3.04( 0.26, 5.82)*** | **1.53(0.29,2.78)*** | 1.6(-0.36, 3.57) | **8.75(3.64,13.86)***** | **7.32( 3.04, 11.60)**** | **1.08(0.77,1.38)***** | **1(0.52, 1.47)**** |
|  | Q1 | Ref | Ref | Ref | Ref | Ref | Ref | Ref | Ref | Ref | Ref | Ref | Ref |
|  | Q2 | **-21.68(-34.50, -8.86)**** | -14.92(-30.64, 0.80) | **-19.14(-30.19, -8.09)**** | **-13.98(-27.88,-0.07)*** | 4.04(-0.55, 8.63) | 3.27(-2.04, 8.57) | 1.95(-1.14,5.04) | 1.88(-1.50, 5.26) | **15.66(2.84,28.49)*** | 7.06( -7.03, 21.15) | **1.5(0.82,2.19)**** | 0.82(-0.01, 1.66) |
|  | Q3 | **-27.08(-37.48,-16.69)***** | **-17.22(-29.92,-4.52)*** | **-24.06(-33.21,-14.92)***** | **-15.53(-26.89,-4.17)*** | 2.28(-1.55, 6.11) | 2.27(-3.05, 7.60) | 0.02(-2.35,2.40) | 1.03(-2.52, 4.58) | **25.03(9.68,40.39)**** | 9.15( -1.88, 20.17) | **2.14(1.37,2.90)***** | **1.09( 0.21, 1.97)*** |
|  | Q4 | **-18.35(-31.75, -4.95)**** | -12.05(-26.67, 2.58) | **-17.11(-27.75, -6.46)**** | -11.47(-23.80, 0.86) | **6.87( 3.07,10.68)***** | **6.78(1.61,11.95)*** | **3.68( 0.74,6.62)*** | **3.89( 0.13, 7.66)*** | **17.24(7.52,26.95)**** | **10.93( 0.17, 21.68)*** | **2.3(1.59,3.01)***** | **1.84( 0.46, 3.23)*** |
| Manganese` | Continues | **-18.92(-33.90,-3.94)*** | -8.16(-21.00, 4.68) | **-15.29(-27.75,-2.83)*** | -6.55(-18.40, 5.29) | 4.34(-0.42,9.09) | 4.84(-1.51,11.19) | 2.42(-1.26,6.10) | 2.37(-3.19, 7.92) | 8.28(-2.92,19.48) | 10.89(-5.04, 26.83) | 0.5(-0.54,1.55) | 0.14(-0.95, 1.24) |
|  | Q1 | Ref | Ref | Ref | Ref | Ref | Ref | Ref | Ref | Ref | Ref | Ref | Ref |
|  | Q2 | -2.28(-19.97,15.40) | 7.97(-13.42,29.36) | -1.13(-16.02,13.76) | 7.08(-10.78,24.93) | 2.99(-0.61,6.60) | 1.67( -2.23, 5.58) | 2.41(-0.04,4.85) | 1.23(-1.19, 3.65) | -3.27(-13.71, 7.17) | -1.13(-14.31, 12.05) | -0.48(-1.06,0.10) | **-0.95(-1.61,-0.30)*** |
|  | Q3 | **-13.9(-26.51,-1.29)*** | -7.05(-26.16,12.05) | **-10.81(-20.99,-0.63)*** | -5.49(-20.77, 9.80) | 1.32(-1.39,4.03) | 2.35(-2.36, 7.06) | 1.05(-0.89,2.98) | 1.62(-1.75, 4.99) | -1.24(-11.86, 9.38) | -0.82(-14.35, 12.71) | 0.27(-0.70,1.23) | 0.08(-0.86, 1.02) |
|  | Q4 | **-13.23(-26.29,-0.18)*** | -5.16(-20.20, 9.87) | -10.58(-21.23, 0.07) | -3.91(-15.88, 8.06) | **4.03( 0.67,7.38)*** | 4.49(-0.30, 9.29) | **2.47( 0.08,4.86)*** | 2.73(-0.77, 6.23) | 5.73(-4.99,16.45) | 2.83(-14.34, 19.99) | 0.59(-0.36,1.53) | 0.41(-0.62, 1.44) |
| Selenium | Continues | -20.08(-49.33,9.17) | -17.63(-57.26,22.00) | -14.57(-38.81,9.68) | -13.75(-46.03,18.54) | -1.85(-12.69,9.00) | -1.97(-15.05,11.12) | 1.03(-7.07,9.14) | 1.56(-7.32,10.43) | -20.81(-49.50,7.87) | -29.13(-59.95, 1.70) | 1.23(-2.61,5.06) | 1.17(-3.04, 5.37) |
|  | Q1 | Ref | Ref | Ref | Ref | Ref | Ref | Ref | Ref | Ref | Ref | Ref | Ref |
|  | Q2 | 2.75(-11.34,16.85) | 0.54(-18.77,19.86) | 2.57( -9.17,14.31) | 0.63(-15.15,16.42) | -0.71(-5.15,3.72) | -0.53( -5.54, 4.47) | -0.19(-3.27,2.89) | 0.08(-3.53,3.70) | -4.85(-17.21, 7.50) | -4.1(-17.62, 9.42) | -0.45(-1.40, 0.49) | -0.07(-1.01, 0.87) |
|  | Q3 | 1.18(-12.09,14.44) | -0.83(-16.83,15.17) | 1.85(-9.40,13.10) | -0.1(-13.74,13.54) | -2.82(-6.14,0.50) | -2.09( -5.70, 1.52) | -1.2(-3.74,1.34) | -0.9(-3.83,2.03) | **-11.24(-20.03,-2.46)*** | -7.77(-20.62, 5.08) | **-0.78(-1.55,-0.01)*** | -0.33(-1.10, 0.44) |
|  | Q4 | -8.1(-18.75, 2.54) | -6.4(-19.47, 6.67) | -6.13(-14.98, 2.71) | -5(-15.63, 5.62) | 0.87(-3.46,5.19) | 0.71( -4.50, 5.91) | 1(-2.43,4.43) | 1.39(-2.38,5.17) | -1.65(-13.37,10.08) | -8.42(-22.12, 5.28) | 0.54(-0.58, 1.65) | 0.42(-0.77, 1.60) |

E2, estradiol; FE2, free estradiol; TT, total testosterone; FT, free testosterone; SHBG, sex hormone-binding globulin; TT/E2, the ratio of TT to E2.

Adjusted for age, race, education, marital status, poverty, body mass index, smoking, alcohol assumption, recreational activity, type of menopause, time since menopause, parity, time of blood sampling and energy intake.

**S Table 4.** Relationship between blood concentrations of lead and sex hormones in post-menopausal women stratified by type of menopause(weighted）

| Type of menopause |  | E2  β(95%CI) | | fE2  β(95%CI) | | TT  β(95%CI) | | fTT  β(95%CI) | | SHBG  β(95%CI) | | TT/E2  β(95%CI) | |
| --- | --- | --- | --- | --- | --- | --- | --- | --- | --- | --- | --- | --- | --- |
|  |  | Crude Model | Adjusted Model | Crude Model | Adjusted Model | Crude Model | Adjusted Model | Crude Model | Adjusted Model | Crude Model | Adjusted Model | Crude Model | Adjusted Model |
| Natural menopause (n=557) | Continues | **-21.29**  **(-28.50,**  **-14.08)**  ******* | **-6.71(-11.91,-1.51)*** | **-18.41**  **(-24.45,**  **-12.37)**  ******* | **-5.99(-10.18,-1.80)*** | 1.87(-0.83,4.57) | 2.2(-1.76, 6.16) | 0.21(-1.53,1.95) | 0.6(-1.96, 3.16) | **16.32(10.10,22.54)***** | **10.19( 1.52, 18.87)*** | **1.87(1.38,2.35)***** | 0.75(-0.09, 1.59) |
|  | Quantile 1 | Ref | Ref | Ref | Ref | Ref | Ref | Ref | Ref | Ref | Ref | Ref | Ref |
|  | Quantile 2 | **-31.79**  **(-45.28,**  **-18.29)**  ******* | **-17.7(-31.88,-3.53)*** | **-27.23**  **(-38.52,**  **-15.93)**  ******* | **-15.75**  **(-27.78,**  **-3.72)*** | 0.68(-3.93,5.30) | 1.89( -2.58, 6.35) | 0.4(-2.87,3.67) | 1.48(-1.98, 4.94) | 6.27(-4.55,17.09) | 0.3(-10.94, 11.54) | **2.16(1.26,3.06)***** | 1.32( 0.20, 2.43)* |
|  | Quantile 3 | **-27.59**  **(-42.65,**  **-12.53)**  ****** | -8.43(-22.99, 6.13) | **-23.7(-36.47,-10.93)**** | -7.84(-19.99, 4.32) | -1(-4.90,2.90) | -0.07( -2.90, 2.77) | -1.09(-3.80,1.62) | -0.05(-2.10, 2.01) | 8.29(-3.31,19.89) | 1.39(-10.50, 13.28) | **1.78(1.04,2.53)***** | 0.42(-0.24, 1.08) |
|  | Quantile 4 | **-36.14**  **(-48.23,**  **-24.06)**  ******* | **-15.98**  **(-26.35,**  **-5.60)*** | **-31.05**  **(-41.23,**  **-20.87)**  ******* | **-14(-22.70,-5.31)*** | 4.77(0.04,9.49)* | 4.93(-0.93,10.79) | 1.57(-1.49,4.63) | 2.12(-1.68, 5.93) | **25.23(13.08,37.38)***** | **13.74( 0.89, 26.59)*** | **3.38(2.45,4.31)***** | **1.85(0.62, 3.09)*** |
| Hysterectomy (n=136) | Continues | **-22.56**  **(-35.31,**  **-9.82)**** | 5.23(-23.14, 33.59) | **-19.43**  **(-29.56,**  **-9.29)**** | 2.28(-20.92, 25.47) | -1.04(-4.97,2.89) | 1.74( -3.68, 7.17) | -1.51(-4.05,1.03) | -0.38(-4.05, 3.30) | **18.08(3.85,32.32)*** | **32.54( 20.28,44.80)***** | **1.31(0.56,2.06)**** | 0.13(-0.85, 1.11) |
|  | Quantile 1 | Ref | Ref | Ref | Ref | Ref | Ref | Ref | Ref | Ref | Ref | Ref | Ref |
|  | Quantile 2 | **-38.16**  **(-67.43, -8.90)*** | -4.44( -31.11, 22.23) | **-32.2(-55.79, -8.62)*** | -5.25(-27.98, 17.49) | 0.03(-9.43,9.49) | 1.73( -4.60, 8.06) | -0.92(-6.96,5.12) | 0.51(-3.68, 4.70) | 13.24  ( -2.30,  28.78) | 9.42( -3.07,21.90) | 1.27(-0.06,2.60) | -0.18(-1.48, 1.13) |
|  | Quantile 3 | **-49.53**  **(-74.54,**  **-24.51)**  ****** | -1(-27.81, 25.82) | **-41.71**  **(-61.98,**  **-21.45)**  ****** | -3.38(-24.78, 18.01) | -1.1(-8.69,6.48) | -0.91( -8.40, 6.58) | -2.27(-7.66,3.13) | -1.4(-6.67, 3.87) | 25.23  (-11.02,  61.49) | 9.42( -3.07,21.90) | **1.99( 0.77,3.20)**** | -0.47(-1.50, 0.57) |
|  | Quantile 4 | **-31.64**  **(-58.12, -5.16)*** | -7.89( -33.57, 17.78) | **-27.58**  **(-48.45, -6.71)*** | -9.28(-30.47, 11.92) | -2.53(-9.72,4.67) | 2.1( -5.94,10.14) | -3.04(-7.75,1.66) | -1(-6.42, 4.42) | **28.97( 9.78,48.16)*** | **46.63( 29.20,64.06)***** | **1.92( 0.72,3.12)**** | 0.77(-0.76, 2.29) |

E2, estradiol; FE2, free estradiol; TT, total testosterone; FT, free testosterone; SHBG, sex hormone-binding globulin; TT/E2, the ratio of TT to E2.

*** :p< 0.001; **: p< 0.01, *: p< 0.05

Adjusted for age, race, education, marital status, poverty, body mass index, smoking, alcohol assumption, recreational activity, time since menopause, parity, time of blood sampling and energy intake.

**S Table 5.** Relationship between blood concentrations of lead and sex hormones in post-menopausal women stratified by BMI (weighted）

| BMI |  | E2  β(95%CI) | | fE2  β(95%CI) | | TT  β(95%CI) | | fTT  β(95%CI) | | SHBG  β(95%CI) | | TT/E2  β(95%CI) | |
| --- | --- | --- | --- | --- | --- | --- | --- | --- | --- | --- | --- | --- | --- |
| <25kg/m2 (n=177) |  | Crude Model | Adjusted Model | Crude Model | Adjusted Model | Crude Model | Adjusted Model | Crude Model | Adjusted Model | Crude Model | Adjusted Model | Crude Model | Adjusted Model |
|  | Continues | **-29.59(-50.60,-8.59)*** | -5.84( -20.40, 8.72) | **-24.53(-41.62,-7.44)*** | -5.4( -16.47, 5.68) | 0.31(-3.53,4.14) | 2.27( -2.73, 7.27) | -0.74  (-3.26,  1.78) | 0.79( -1.96, 3.54) | **18.6(8.14,29.07)**** | 15.23(-1.80, 32.27) | **2.54(1.68,3.39)***** | 1.71( 0.25, 3.16)* |
|  | Quantile 1 | Ref | Ref | Ref | Ref | Ref | Ref | Ref | Ref | Ref | Ref | Ref | Ref |
|  | Quantile 2 | **-63.06(-104.94,-21.19)**** | **-47.88( -93.72, -2.03)*** | **-52.27(-85.57,-18.96)**** | **-40.16(-77.10,-3.23)*** | 0.02( -8.66,8.71) | -1.1( -7.50, 5.29) | -1.02  (-7.21,  5.17) | -1.38( -5.78, 3.02) | 18.97(-11.11,49.04) | 9.05(-19.91,38.02) | **2.89(0.92,4.87)*** | 1.04(-0.95, 3.04) |
|  | Quantile 3 | **-66.59(-105.24,-27.93)**** | -23.83( -63.02, 15.36) | **-55.33(-86.36,-24.30)**** | -21.73(-52.53, 9.08) | -3.23(-11.23,4.76) | -3.96(-12.23, 4.31) | -3.98  (-8.53,  0.58) | -3.76( -8.65, 1.13) | 19.92( -2.84,42.68) | 11.33(-15.99,38.65) | **3.52(1.68,5.35)**** | 1.55(-0.33, 3.42) |
|  | Quantile 4 | **-67.01(-107.22,-26.81)**** | -24.98( -53.94, 3.99) | **-55.17(-87.68,-22.66)**** | -21.73(-44.33, 0.87) | 1.4( -6.24,9.05) | 3.01( -5.14,11.17) | -0.62  (-5.77,  4.53) | 0.53( -3.93, 4.99) | **33.07( 13.45,52.69)**** | 26.73( -0.76,54.23) | **4.8(3.14,6.46)***** | **2.81( 0.36, 5.25)*** |
| 25~29.9kg/m2 (n=186) | Continues | **-17.17(-31.95,-2.38)*** | 3.37(-7.34, 14.07) | **-14.44(-26.83,-2.04)*** | 2.56( -6.61, 11.73) | 1.18(-4.00,6.36) | 2.58( -0.72, 5.88) | 0.05(-2.83,2.93) | -0.18( -2.48, 2.12) | 10.71(-5.09,26.51) | **19.02( 7.63,30.41)**** | **1.21(0.27,2.15)*** | -0.11(-0.83,0.61) |
|  | Quantile 1 | Ref | Ref | Ref | Ref | Ref | Ref | Ref | Ref | Ref | Ref | Ref | Ref |
|  | Quantile 2 | -25.27(-45.54,-5.01) | **-16.38(-29.38, -3.38)*** | **21.27(-38.19,-4.35)*** | **-14.03(-25.19, -2.86)*** | -2.2(-8.88, 4.47) | 0.72( -5.64, 7.09) | -0.7(-5.04,3.64) | 0.48( -4.04, 5.00) | -6.17(-38.55,26.22) | 2.93(-11.76,17.61) | **1.61( 0.26,2.96)*** | **1.14(0.02,2.27)*** |
|  | Quantile 3 | -16.85(-47.96,14.26) | -4.33(-30.14, 21.49) | -12.85(-40.32,14.61) | -2.42(-25.51, 20.68) | -2.18(-9.12, 4.77) | -1.12(-6.43, 4.20) | -0.71  (-5.03,  3.62) | -1.27( -5.28, 2.74) | -5.74(-36.94,25.46) | 4.73(-10.68,20.15) | 1.07(-0.49,2.63) | -0.1(-1.21,1.01) |
|  | Quantile 4 | **-28.98(-50.61,-7.36)*** | -11.19(-30.47, 8.10) | **-24.48(-42.48,-6.48)*** | -9.67(-25.88, 6.54) | 3.06(-4.80,10.93) | 6.03(-0.11,  12.16) | 1.07(-3.04,5.18) | 1.51( -2.41, 5.42) | 7.63(-23.10,38.36) | **24.41( 10.97,37.84)**** | **2.11(0.28,3.94)*** | **0.94(-0.36,2.24)*** |
| ≥30kg/m2 (n=328) | Continues | **-19.47(-28.51,-10.43)***** | 1.81( -7.62,11.23) | **-17.44(-25.17,-9.72)***** | 0.17( -7.52, 7.86) | 2.44(-1.88,6.75) | 4.42(-1.12, 9.97) | 0.84(-2.04,3.72) | 1.83( -1.65, 5.32) | **11.43(6.08,16.79)***** | **18.43( 10.17,26.69)**** | **1.15(0.60,1.69)***** | 0.78(-0.19, 1.74) |
|  | Quantile 1 | Ref | Ref | Ref | Ref | Ref | Ref | Ref | Ref | Ref | Ref | Ref | Ref |
|  | Quantile 2 | **24.29(-38.37,-10.22)**** | -5.31(-31.92,21.30) | **21.33(-33.61, -9.05)**** | -6.58(-29.56,16.39) | 2.25(-3.22, 7.72) | 3.72( -2.36, 9.80) | 1.25(-2.54,5.03) | 2.09( -2.18, 6.37) | 6.51(-1.36,14.37) | 10.46( -1.32,22.24) | **1.47(0.33,2.61)*** | 1.15(-0.13, 2.44) |
|  | Quantile 3 | **-27.4(-41.75,-13.05)***** | -1.63(-22.32,19.05) | **-24.92(-36.42,-13.41)***** | -4(-20.28,12.28) | 0.75(-3.77, 5.27) | 3.55( -2.31, 9.40) | 0.21(-3.14,3.57) | 2( -2.36, 6.36) | **8.53( 0.20,16.86)*** | 14.13( -2.86,31.12) | **1.02(0.59,1.46)***** | **0.8(0.01, 1.59)*** |
|  | Quantile 4 | **-26.29(-45.03, -7.55)*** | -1.69(-23.26,19.89) | **-23.66(-39.41, -7.91)**** | -3.48(-21.22,14.27) | 4.94(-2.86,12.74) | 7.21( -2.72,17.15) | 1.88(-3.29,7.06) | 3.25( -3.19, 9.70) | **19.85( 9.75,29.94)***** | **26.39( 11.25,41.54)*** | **2.05(1.08,3.02)**** | 1.66( 0.43, 2.89)* |

E2, estradiol; FE2, free estradiol; TT, total testosterone; FT, free testosterone; SHBG, sex hormone-binding globulin; TT/E2, the ratio of TT to E2.

Adjusted for age, race, education, marital status, poverty, smoking, alcohol assumption, recreational activity, type of menopause, time since menopause, parity, time of blood sampling and energy intake.

**S Table 6**. Relationship Between blood concentrations of cadmium and sex hormones in post-menopausal women stratified by type of menopause(weighted）

| Type of menopause |  | E2  β(95%CI) | | fE2  β(95%CI) | | TT  β(95%CI) | | fTT  β(95%CI) | | SHBG  β(95%CI) | | TT/E2  β(95%CI) | |
| --- | --- | --- | --- | --- | --- | --- | --- | --- | --- | --- | --- | --- | --- |
|  |  | Crude Model | Adjusted Model | Crude Model | Adjusted Model | Crude Model | Adjusted Model | Crude Model | Adjusted Model | Crude Model | Adjusted Model | Crude Model | Adjusted Model |
| Natural menopause (n=557) | Continues | **-7.98(-14.98,-0.97)*** | -7.26(-17.42, 2.90) | **-7.74(-13.44,-2.04)*** | -7.1(-16.06, 1.86) | **3.35(1.63,5.08)***** | 2.83( -0.13, 5.80) | **1.71(0.33,3.08)*** | 1.59(-0.55, 3.72) | **8.76(2.66,14.86)*** | **6.15(0.81,11.49)**  ***** | **1.16(0.81,1.51)***** | **1.07(0.48, 1.66)**** |
|  | Quantile 1 | Ref | Ref | Ref | Ref | Ref | Ref | Ref | Ref | Ref | Ref | Ref | Ref |
|  | Quantile 2 | **-23.16**  **(-38.34, -7.99)**** | -16.26(-33.65, 1.13) | **-20.29**  **(-33.48, -7.10)**** | -14.94  (-30.69, 0.81) | **4.88(0.35, 9.41)*** | 1.91( -3.85, 7.66) | 2.41(-0.70,5.53) | 1.27(-2.55, 5.09) | **15.97( 0.99,30.95)*** | 5.22( -9.54, 19.98) | **1.58(0.78,2.38)**** | 0.94(-0.04, 1.92) |
|  | Quantile 3 | **-26.85**  **(-41.44,**  **-12.26)**  ******* | -14.76(-29.84, 0.32) | **-23.78**  **(-36.40,**  **-11.16)**  ******* | **-13.48**  **(-26.91,**  **-0.06)*** | 2.11(-1.29, 5.52) | 1.12( -4.53, 6.77) | -0.04(-2.28,2.21) | 0.45(-3.34, 4.23) | **23.82(13.01,34.62)***** | 7.92( -3.19, 19.04) | **2.37(1.51,3.23)***** | **1.22(0.27, 2.16)*** |
|  | Quantile 4 | -15.58(-31.62, 0.45) | -12.58(-28.39, 3.23) | **-14.86**  **(-27.80, -1.92)*** | -11.78(-25.12, 1.56) | **8.2(3.82,12.58)***** | **7.63(1.36,13.91)*** | **4.62(1.36,7.88)*** | **4.92(0.29, 9.55)*** | **16.62( 5.04,28.20)*** | 8.02(-1.32, 17.35) | **2.27(1.50,3.04)***** | **1.96(0.59, 3.34)*** |
| Hysterectomy (n=136) | Continues | -12.24(-24.61,0.13) | -15.13  (-31.29, 1.03) | **-10.85**  **(-20.63,**  **-1.08)*** | -13.26(-26.46, -0.06)* | 2.05(-1.49,5.59) | 2.77(-0.02,5.56) | 0.91(-1.43,3.25) | 0.95(-0.99, 2.89) | 8.68(-5.83,23.18) | 10.98( -2.68, 24.65) | **0.8(0.12,1.48)*** | **0.75(0.00, 1.51)*** |
|  | Quantile 1 | Ref | Ref | Ref | Ref | Ref | Ref | Ref | Ref | Ref | Ref | Ref | Ref |
|  | Quantile 2 | -14.52(-36.27, 7.24) | -19.42(-39.37, 0.52) | -13.56  (-31.75, 4.64) | -17.72(-34.00, -1.45)* | 2.65(-4.81,10.12) | **5.12( 1.29, 8.96)*** | 0.93(-3.92,5.78) | 2.43(-0.17, 5.03) | 14.35( -4.51,  33.21) | 9.71(-11.80, 31.21) | **1.14(0.29,1.99)*** | 0.6(-0.24, 1.45) |
|  | Quantile 3 | -27.71(-54.29,-1.12)* | -34.67(-60.19, -9.15)* | **-24.94**  **(-46.38,**  **-3.51)*** | -30.41(-51.81, -9.02)* | 1.66(-9.40,12.72) | 1.42( -4.52, 7.37) | -0.68(-6.81,5.44) | 0.49(-3.51, 4.48) | 29.93  (-19.54,  79.41) | 6.06(-11.78, 23.90) | 1.19(-0.29,2.68) | **1.24( 0.09, 2.39)*** |
|  | Quantile 4 | -29.13(-53.40,-4.87)* | 1.76(-27.81, 31.34) | **-25.92**  **(-45.16,**  **-6.68)*** | -0.32(-23.76, 23.11) | 3.23(-5.08,11.54) | 5.74(-0.73,  12.21) | 1.04(-4.45,6.53) | 1.01(-2.98, 5.00) | 20.25( -5.56,  46.05) | 29.56(-3.29, 62.42) | **2.3( 0.36,4.24)*** | 1.33(-0.47, 3.13) |

E2, estradiol; FE2, free estradiol; TT, total testosterone; FT, free testosterone; SHBG, sex hormone-binding globulin; TT/E2, the ratio of TT to E2.

*** :p< 0.001; **: p< 0.01, *: p< 0.05

Adjusted for age, race, education, marital status, poverty, body mass index, smoking, alcohol assumption, recreational activity, time since menopause, parity, time of blood sampling and energy intake.

**S Table 7.** Relationship between blood concentrations of cadmium and sex hormones in post-menopausal women stratified by BMI (weighted）

| BMI |  | E2  β(95%CI) | | fE2  β(95%CI) | | TT  β(95%CI) | | fTT  β(95%CI) | | SHBG  β(95%CI) | | TT/E2  β(95%CI) | |
| --- | --- | --- | --- | --- | --- | --- | --- | --- | --- | --- | --- | --- | --- |
| <25kg/m2 (n=177) |  | Crude Model | Adjusted Model | Crude Model | Adjusted Model | Crude Model | Adjusted Model | Crude Model | Adjusted Model | Crude Model | Adjusted Model | Crude Model | Adjusted Model |
|  | Continues | -4.66(-26.47,17.16) | -3.25(-22.34, 15.83) | -4.57(-21.74,12.61) | -3.4( -18.81, 12.01) | 4.07(1.13,7.00)* | 2.35( -0.72, 5.42) | 1.53(-0.17,3.23) | 1.09( -0.87, 3.06) | **18.12(8.09,28.15)***** | **13.02( 2.95,23.08)*** | **1.15(0.07,2.23)*** | 0.94(-0.10, 1.98) |
|  | Quantile 1 | Ref | Ref | Ref | Ref | Ref | Ref | Ref | Ref | Ref | Ref | Ref | Ref |
|  | Quantile 2 | **-57.61(-101.30,-13.92)*** | -28.15( -62.75, 6.45) | **-47.05(-82.10,-12.01)*** | -28.15( -62.75, 6.45) | 3.73(-2.74,10.21) | -0.47( -6.58, 5.65) | 2.03(-2.93,7.00) | 0.56( -3.44, 4.56) | 21.82(-2.67,46.31) | -13.22(-34.91, 8.47) | **3.89(1.96,5.82)***** | 1.98(-0.18, 4.13) |
|  | Quantile 3 | **-55.46( -99.95,-10.97)*** | -29.66( -66.32, 7.00) | **-46.03(-81.88,-10.18)*** | -29.66( -66.32, 7.00) | 3.01(-0.18, 6.20) | 0.35( -6.52, 7.23) | 0.01(-2.30,2.32) | 0.61( -3.88, 5.10) | **42.01(16.96,67.05)**** | 11.56(-17.95,41.07) | **3.37(1.59,5.14) ***** | 1.23(-0.70, 3.17) |
|  | Quantile 4 | -30.74( -79.62, 18.15) | -26.8( -73.09, 19.49) | -26.36(-65.29, 12.58) | -26.8( -73.09, 19.49) | 7.66( 1.78,13.54)* | 2.52( -4.60, 9.65) | 2.88(-0.36,6.11) | 1.09( -3.69, 5.86) | **38.72(19.19,58.26)***** | 14.49(-11.12,40.09) | **3.39(1.57,5.21) ***** | 2.17(-0.27, 4.60) |
| 25~29.9kg/m2 (n=186) | Continues | **-12.02(-23.87,-0.18)*** | -14.67(-31.08, 1.73) | -10.96(-21.98,0.06) | -13.48(-28.72, 1.75) | 3.28(-0.08,6.63) | 0.64( -4.17, 5.44) | 1.98(-0.60,4.57) | 0.24( -2.78, 3.26) | 2.34(-5.41,10.08) | 4.56( -6.32,15.44) | **0.89(0.47,1.31)***** | 0.8(-0.09,1.69) |
|  | Quantile 1 | Ref | Ref | Ref | Ref | Ref | Ref | Ref | Ref | Ref | Ref | Ref | Ref |
|  | Quantile 2 | -20.08(-48.72,8.57) | -19.72(-50.36, 10.93) | -18.45(-44.44,7.54) | -18.62(-47.36, 10.12) | 2.64(-4.12, 9.40) | -1.57(-10.90, 7.77) | 0.48(-3.95, 4.91) | -1.5( -7.78, 4.79) | 20.07(-4.05,44.18) | 10.62( -9.44,30.67) | 0.99(-0.17,2.16) | 0.62(-1.05,2.29) |
|  | Quantile 3 | -23.19(-50.68,4.29) | -17.19(-44.18, 9.81) | -21.22(-46.34,3.91) | -16.13(-41.03, 8.77) | 0.97(-4.63, 6.58) | 0.65( -6.61, 7.91) | -0.8(-4.47, 2.87) | 0.3( -4.32, 4.92) | 16.26(-0.59,33.12) | 5.87(-12.26,24.00) | 1.37(-0.06,2.81) | 0.93(-0.59,2.46) |
|  | Quantile 4 | -23.69(-51.38,4.01) | -17.16(-38.40, 4.08) | -21.66(-46.95,3.64) | -15.72(-34.61, 3.18) | 7.13(-1.40,15.66) | 1.77( -9.42,12.96) | 4.25(-2.32,10.82) | 0.58( -7.23, 8.40) | 7.06(-9.15,23.26) | 7.06(-18.27,32.40) | 1.9( 0.53,3.28)* | 1.24(-0.97,3.46) |
| ≥30kg/m2 (n=328) | Continues | **-9.79(-17.85,-1.74)*** | -7.67(-17.93, 2.59) | **-9.25(-15.89,-2.61)*** | -7.42(-15.92, 1.08) | 2.66(-0.49,5.82) | **4.94(0.88, 8.99)*** | 1.64(-0.61,3.89) | **2.89(0.07, 5.71)*** | 3.39(-1.99,8.78) | **6.86( 1.04,12.69)*** | **0.92(0.34,1.49)**** | **1.21(0.26, 2.16)*** |
|  | Quantile 1 | Ref | Ref | Ref | Ref | Ref | Ref | Ref | Ref | Ref | Ref | Ref | Ref |
|  | Quantile 2 | -9.12(-26.52, 8.28) | -7.29(-27.14,12.56) | -8.93(-23.86, 6.00) | -7.63(-23.77, 8.51) | 5.28(-0.01,10.58) | **6.79(0.19,13.38)*** | 3.15(-0.46,6.77) | 4.02( -0.43, 8.47) | 7.74(-0.49,15.96) | 10(-3.47,23.48) | **0.7(0.19,1.22)*** | 0.56(-0.33, 1.45) |
|  | Quantile 3 | **-18.35(-32.57,-4.14)*** | -10.59(-24.26, 3.09) | **-16.97(-29.19,-4.75)*** | -9.69(-20.99, 1.61) | 3.51(-3.68,10.70) | 4.84(-4.23,13.91) | 1.53(-3.10,6.15) | 2.73( -2.98, 8.45) | **13.15( 2.59,23.71)*** | 8.05(-6.95,23.05) | **1.63(0.33,2.93)*** | **1.44( 0.03, 2.84)*** |
|  | Quantile 4 | -16.18(-33.91, 1.56) | -10.4(-33.67,12.87) | **-15.21(-30.24,-0.19)*** | -10.19(-29.65, 9.27) | **6.81( 1.12,12.51)*** | **10.82(4.14,17.50)*** | **4.62( 0.60,8.64)*** | **6.83( 2.47,11.20)*** | 3.96(-6.86,14.78) | 9.59( -3.26,22.44) | **1.76(0.50,3.02)*** | 2.14(-0.29, 4.57) |

E2, estradiol; FE2, free estradiol; TT, total testosterone; FT, free testosterone; SHBG, sex hormone-binding globulin; TT/E2, the ratio of TT to E2.

Adjusted for age, race, education, marital status, poverty, smoking, alcohol assumption, recreational activity, type of menopause, time since menopause, parity, time of blood sampling and energy intake

#
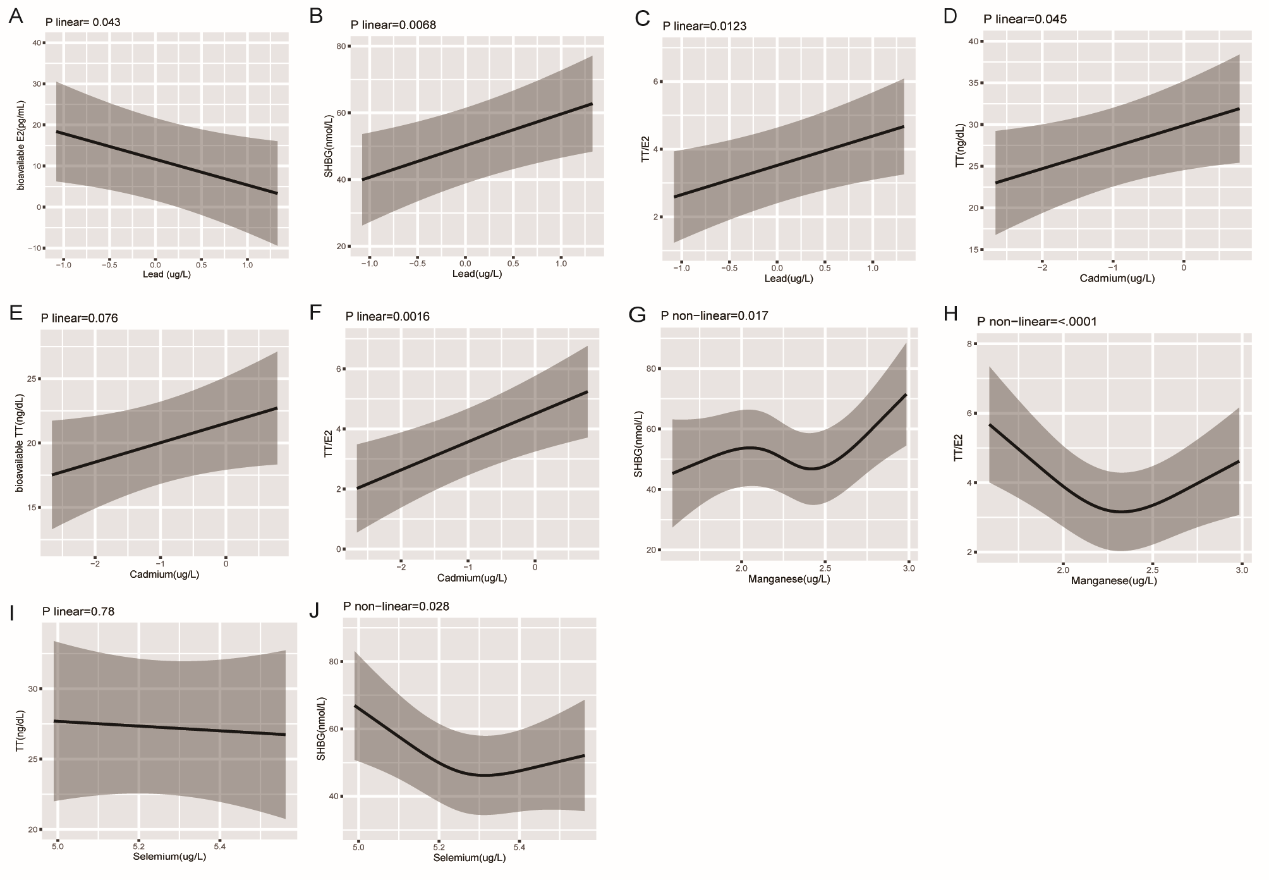
Supplementary Figures

**S-Figure 1**. Restricted cubic splines and linear models of association between blood concentration of heavy metals and sex hormones in women who underwent natural menopause

TT, total testosterone. Models stratified by type of menopause were adjusted for all covariates except for type of menopause.


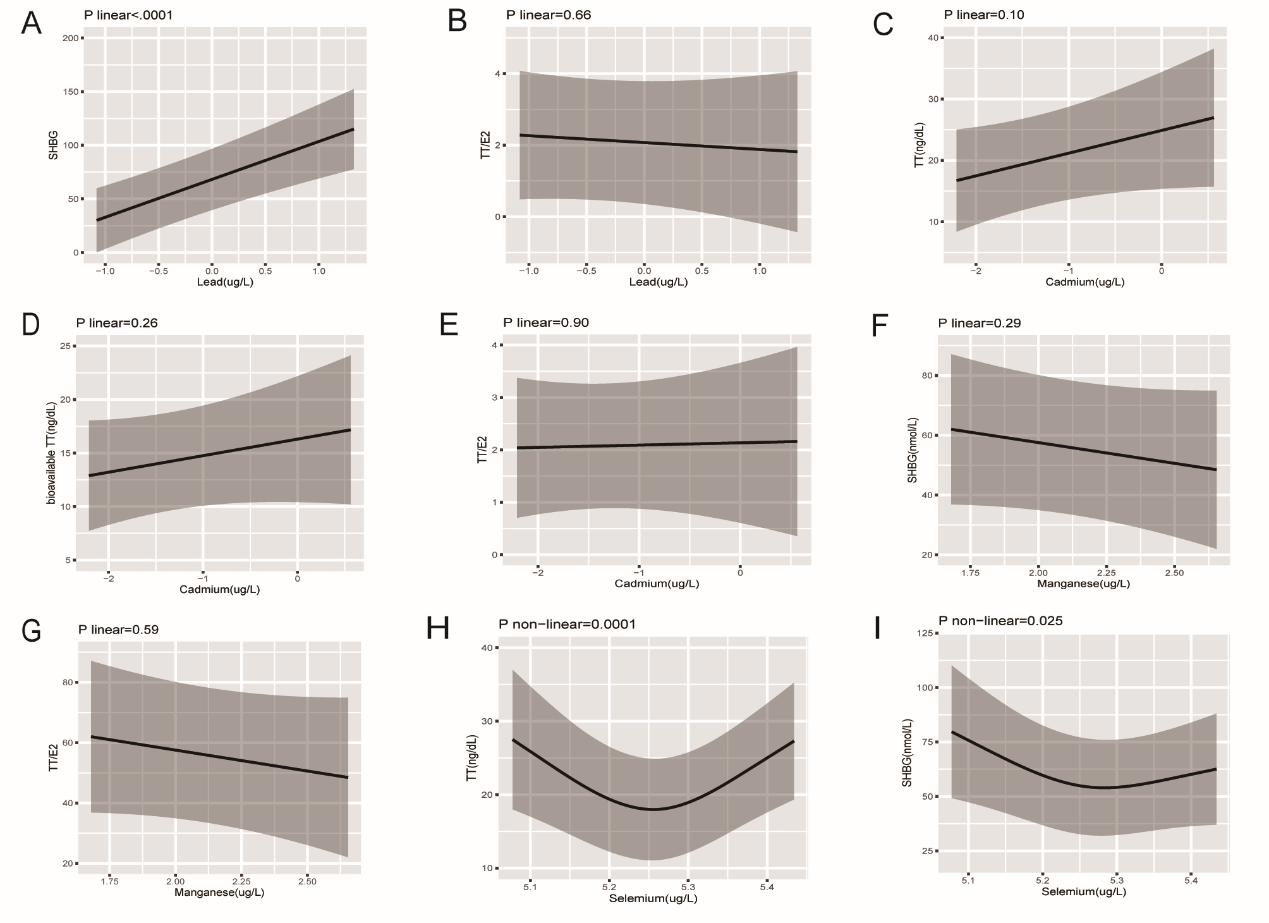


**S-Figure 2.** Restricted cubic splines and linear models of association between blood concentration of heavy metals and sex hormones in women who underwent hysterectomy

TT, total testosterone. Models stratified by type of menopause were adjusted for all covariates except for type of menopause.


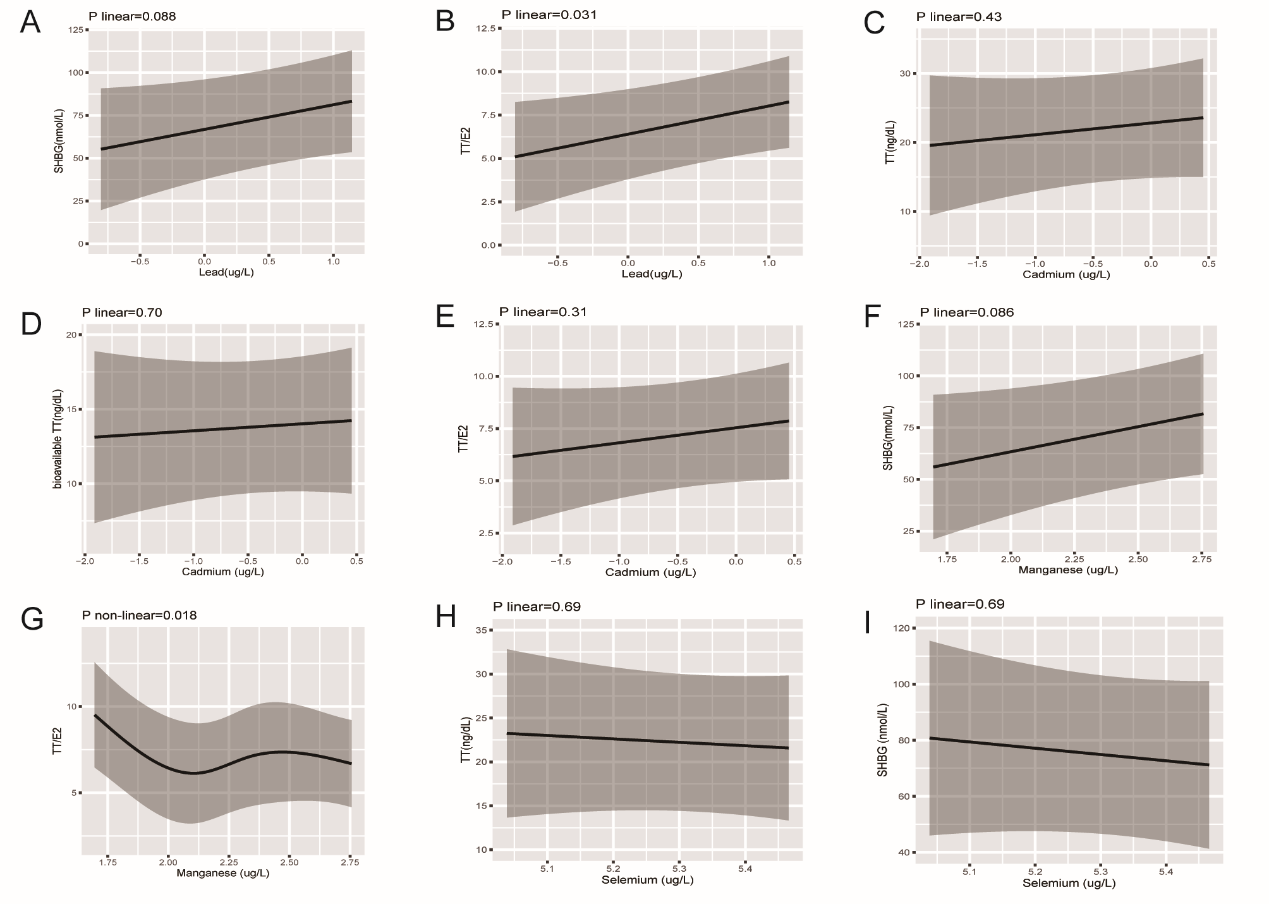


**S-Figure 3**. Restricted cubic splines and linear models of association between blood concentration of heavy metals and sex hormones in women whose BMI was less than <25kg/m2

TT, total testosterone. Models stratified by body mass index were adjusted for all covariates except for body mass index.


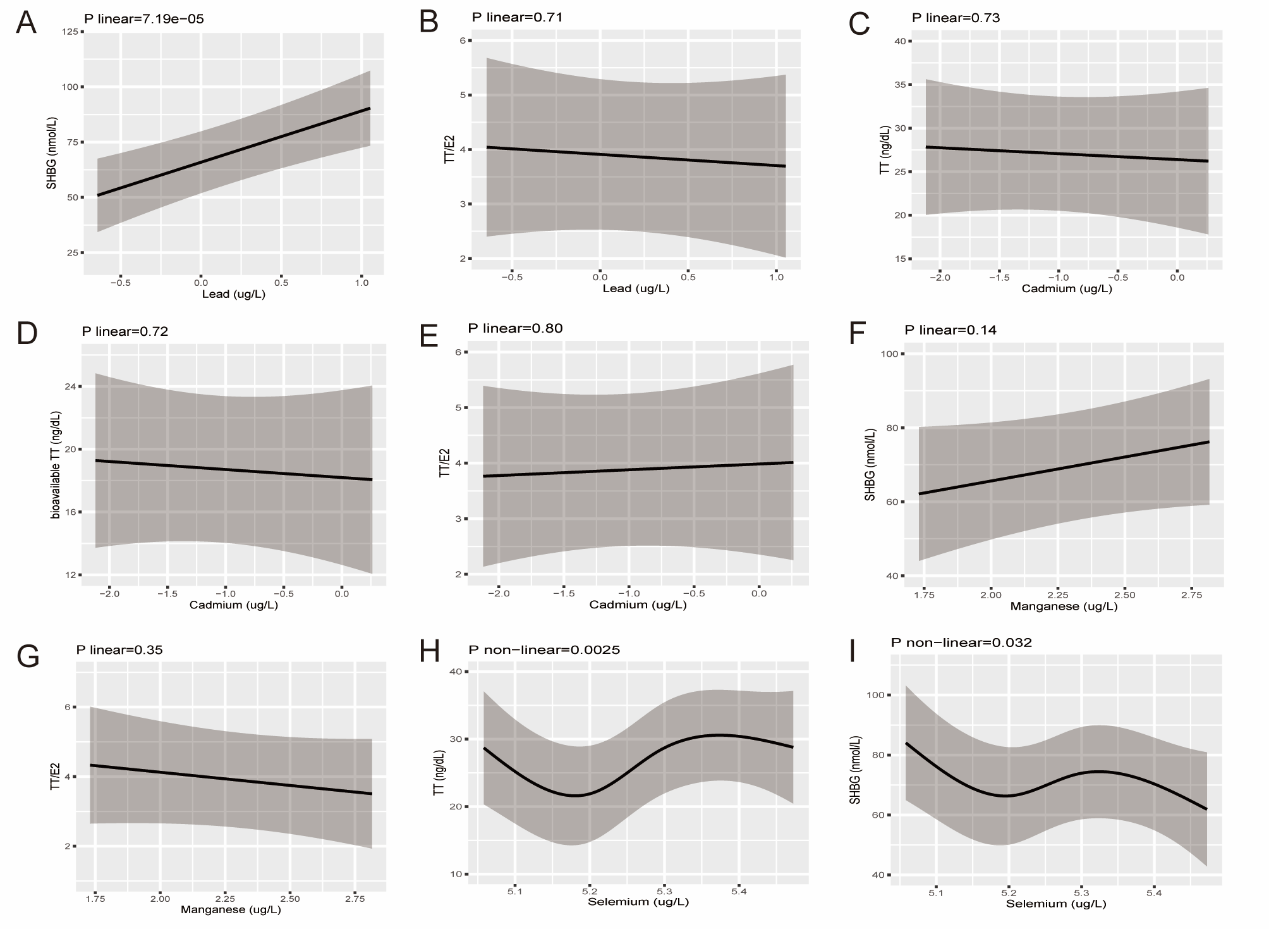


**S-Figure 4.** Restricted cubic splines and linear models of association between blood concentration of heavy metals and sex hormones in women whose BMI was between 25~29.9kg/m2

TT, total testosterone. Models stratified by body mass index were adjusted for all covariates except for body mass index.


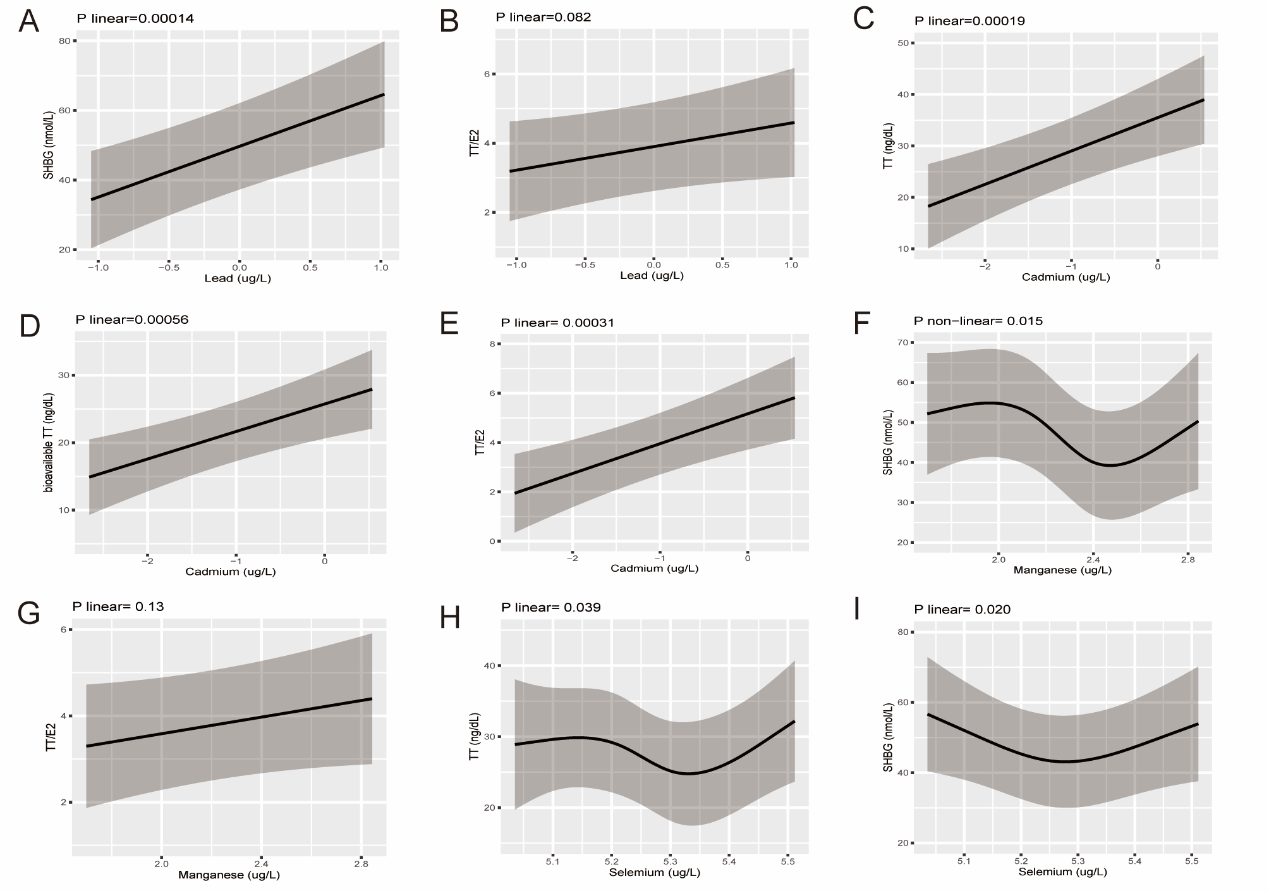


**S-Figure 5.** Restricted cubic splines and linear models of association between blood concentration of heavy metals and sex hormones in women whose BMI was greater than 30kg/m^2^

TT, total testosterone. Models stratified by body mass index were adjusted for all covariates except for body mass index.
